# Activating intramolecular singlet exciton fission by altering π-bridge flexibility in perylene diimide trimers for organic solar cells†

**DOI:** 10.1039/d0sc03271a

**Published:** 2020-08-07

**Authors:** Benedetta Carlotti, Ifeanyi K. Madu, Hyungjun Kim, Zhengxu. Cai, Hanjie Jiang, Angelar K. Muthike, Luping Yu, Paul M. Zimmerman, Theodore Goodson

**Affiliations:** Department of Chemistry, University of Michigan Ann Arbor MI 48109 USA tgoodson@umich.edu; Department of Chemistry Biology and Biotechnology, University of Perugia via Elce di Sotto n.8 06123 Perugia Italy; Department of Chemistry, Incheon National University Incheon 22012 Republic of Korea; Department of Chemistry, The James Franck Institute, The University of Chicago 929 East 57th Street Chicago IL 60637 USA

## Abstract

In this study, two analogous perylene diimide (PDI) trimers, whose structures show rotatable single bond π-bridge connection (twisted) *vs.* rigid/fused π-bridge connection (planar), were synthesized and investigated. We show *via* time resolved spectroscopic measurements how the π-bridge connections in A–π–D–π–A–π–D–π–A multichromophoric PDI systems strongly affect the triplet yield and triplet formation rate. In the planar compound, with stronger intramolecular charge transfer (ICT) character, triplet formation occurs *via* conventional intersystem crossing. However, clear evidence of efficient and fast intramolecular singlet exciton fission (iSEF) is observed in the twisted trimer compound with weaker ICT character. Multiexciton triplet generation and separation occur in the twisted (flexible-bridged) PDI trimer, where weak coupling among the units is observed as a result of the degenerate double triplet and quintet states, obtained by quantum chemical calculations. The high triplet yield and fast iSEF observed in the twisted compound are due not only to enthalpic viability but also to the significant entropic gain allowed by its trimeric structure. Our results represent a significant step forward in structure–property understanding, and may direct the design of new efficient iSEF materials.

## Abstract 2

We show *via* time resolved spectroscopy that triplet formation proceeds *via* intersystem crossing in a rigid-bridged perylene diimide trimer and *via* efficient and fast intramolecular singlet exciton fission in the analogous flexible-bridged trimer.

## Graphical abstract

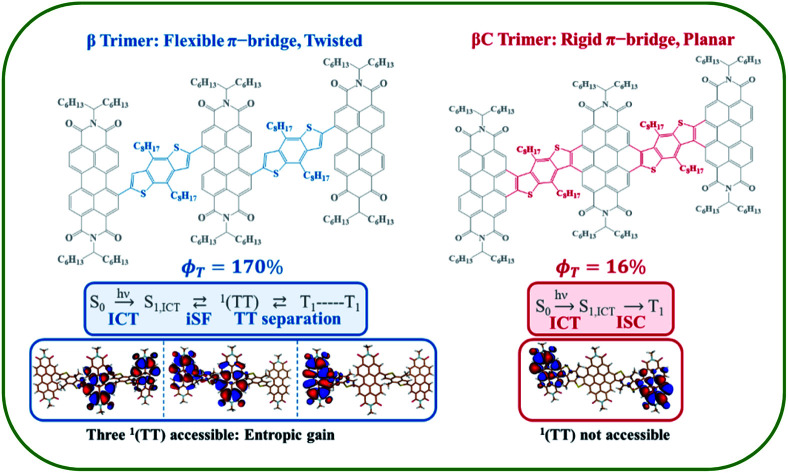

## Introduction

The scientific benefits and applications of understanding the dynamics of multiexciton triplet generation in organic chromophores cannot be overemphasized. One benefit is evaluating the actual potential impact singlet exciton fission (SEF) has on improving the power conversion efficiency (PCE) of organic solar cells.^1^ The effect of SEF on the device photocurrent has been demonstrated by means of magnetic field dependent measurements in literature reports.^2–4^ Another benefit is to account for the excess absorption energy used to generate singlet electron–hole pairs often lost as heat.^5^ Finally, understanding the dynamics of multiexciton generation aids in the careful design and synthesis of selective organic chromophores with high SEF yields, to be used in photovoltaic devices or photocatalytic cells, for the generation of more photocurrent.^6–8^ The ability to advance our insight is limited by the number of materials capable of undergoing SEF.^9,10^ A lot of focus has been placed on acenes (mostly tetracene, pentacene) since the discovery of SEF in anthracene crystals.^7,11–17^ There are relatively fewer SEF studies on perylene diimides (PDIs),^18,19^ which are mostly used as electron acceptors in non-fullerene photovoltaic devices, in comparison to acenes. Understanding the science and mechanism by which PDI acceptors themselves exhibit SEF can be beneficial in avoiding the extra layer to be taken up by a “SEF sensitizer” in an actual photovoltaic device, reducing the complexity, cell thickness, and greatly improving the absorption of solar photons.

Intermolecular SEF (xSEF) has been observed in solid state aggregates of PDI derivatives.^18–22^ High rate of xSEF has often been associated to highly-ordered crystalline systems in comparison to their amorphous counterpart. This has been associated with a SEF assisted process – crystal lattice vibration.^23^ The ordered chromophores have to be in close proximity and achieve a slip-stacked or herringbone dimeric structure. This leads to them having weaker interchromophore (excitonic) interactions.^24^ In these solid state films, triplet formation is significantly influenced by the morphology and crystal packing, which are usually difficult to control. Hence, for devices made with xSEF chromophores where solid-state packing interactions are crucial, slight perturbations can have a drastic effect on the rate and yield of xSEF. This limits the understanding of the underlying key factors affecting the rate and efficiency of singlet fission in xSEF materials. To this regards, a more suitable approach would be intramolecular singlet exciton fission (iSEF).^13,25–29^ Materials capable of iSEF can overcome these challenges because each molecule undergoes SEF *via* through-bond interactions in multichromophoric systems – that is, not depending on intermolecular orientation, intermolecular coupling, or through-space interactions.

Donor–acceptor configuration, which induces an intramolecular charge transfer character, is a molecular design strategy for iSEF molecules.^10^ Another strategy involves the covalent coupling of two xSEF chromophores where the triplet yield has been reported to be affected by the conjugation between the two chromophores.^14,15,30–32^ There are also reports about the effects of through–bond proximity between the chromophores on the triplet production. Campos *et al.*^13^ gave a very detailed account for pentacene dimers where the proximity of the pentacene moieties and the extent of conjugation was varied using (oligo) phenylene spacers. It was suggested that the rate of singlet fission (rate of triplet production) and the rate of recombination of the two triplets could be controlled by using spacers of varying length. In another study, Thompson *et al.*^17^ looked at how connecting two SEF chromophores to a bridge moiety at its *ortho*, *meta* or *para* position influences the through–bond and through-space contributions to the coupling of the compound. Intramolecular SEF was observed only in the *ortho* and *para* systems, not in the *meta*; and this was associated to the lack of effective conjugation, hence very weak coupling, causing predominantly radiative decay of the excited state. However, all these studies were reported for acene dimers for the end purpose of sandwiching them with organic photovoltaic (OPV) active layer materials. There are little to no studies about the effects of the π-bridge in multichromophoric OPV active layer materials themselves, *e.g.* PDIs, in tuning the iSEF rate or in activating/deactivating iSEF.

The aim of this study is to investigate the unique role of the π-bridge in allowing or inhibiting triplet production (rate and yield) in oligomeric multichromophoric PDI systems. In few recent literature reports,^15–17^ structural flexibility of the covalently linked units has been proposed to be crucial in activating SEF. However, these studies lack a direct comparison with rigidly bridged units of the same chromophore in order to isolate the effects of π-bridge flexibility. This is what our current investigation seeks to illuminate – the impact of the π-bridge rotation *vs.* rigidity on the dynamics of triplet exciton formation, and the triplet production efficiency. Most literature studies involve dimers. Investigations about oligomeric structures, with more than two chromophores attached linearly, are very few. In the development of iSEF-OPV materials using a strong acceptor-strong donor configuration,^10^ the role of the flexibility/rigidity of the π-bridge in influencing the triplet production rate and efficiency, has not been investigated.

**Chart 1:**
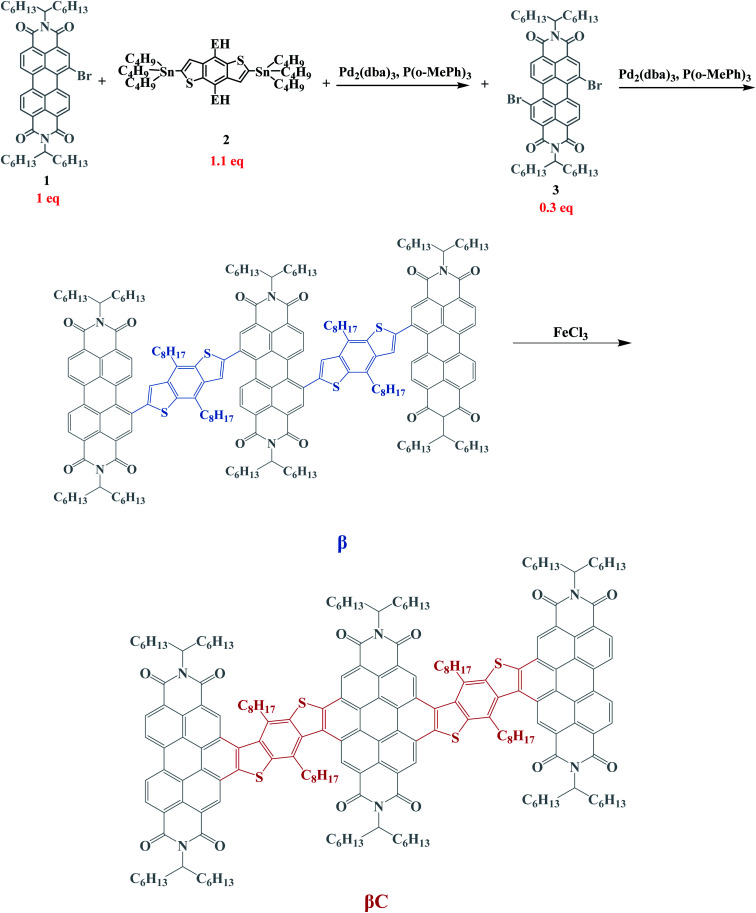
Molecular structures of the investigated trimers.

In this work, two PDI trimers with push–pull character were synthetized (see Chart 1). For each molecule, the unit PDI electron acceptor moieties are bridged at the beta (**β**) position(s) with benzodithiophene (BDT) electron donor moieties, forming an A–D–A–D–A assembly. The connections between the donor and the acceptor moieties were realized *via* single bonds in the **β** compound, and through ring cyclization in the **βC** compound (color coded in the structures in Chart 1). This results in the single-bond-bridged **β** compound having a twisted PDI–BDT–PDI structure (dihedral angles ∼ 55°), and the cyclized **βC** compound achieving a planar PDI–BDT–PDI structure (dihedral angles ∼ 0°). These two compounds show different triplet production dynamics owing to their respective degree of electronic coupling. The photoinduced dynamics of triplet production – *via* iSEF or regular intersystem crossing (ISC) – was thoroughly investigated with a variety of time resolved spectroscopic techniques, employing both femtosecond and nanosecond time resolution while probing both excited state absorption and emission. The experimental spectroscopic study was carried out in a joint effort with theoretical calculations to further elucidate the excited state deactivation mechanism of the two compounds.

## Results

### Steady–state and two–photon absorption measurements

**Fig. 1:**
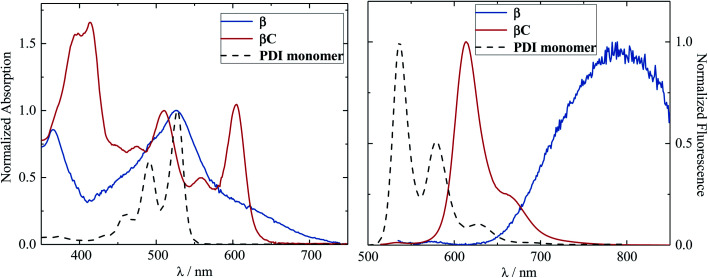
Normalized absorption and emission spectra of the trimers in chloroform.

**Fig. 2:**
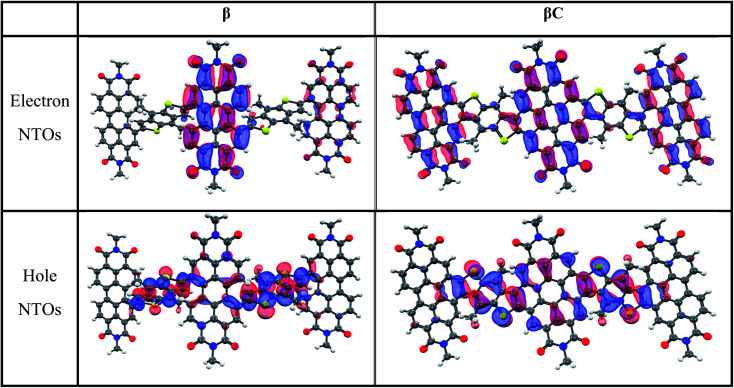
Natural transition orbitals for the S_0_ geometry (S_0_ → S_1_ transition) of the trimers (isodensity = 0.05. Color scheme; hydrogen—white, carbon—black, nitrogen—blue, oxygen—red, sulfur—yellow).

The steady-state absorption and emission spectra of the investigated trimers are shown in Fig. 1. These electron acceptor compounds are excellent light absorbers with sizable molar extinction coefficients, *ca.* 60 000–70 000 M^−1^ cm^−1^ (Table 1). Their broad absorption spectra extend in a region complementary to that of electron donors employed in OPV devices. Both the absorption and emission spectra of **β** appear to be less structured in comparison to those of **βC**. The structured spectra and small Stokes shift (Fig. 1/Table 1) of the **βC** compound reflect its molecular rigidity. The emission spectrum of **β** is extremely broad and its peak is significantly red shifted in comparison to the emission peak of **βC**. The extremely broad emission spectrum and the large Stokes shift suggest a drastic rearrangement of this flexible molecule in the excited state.^33,34^ The theoretical calculations indeed reveal that the **β** compound has a twisted PDI–BDT–PDI structure (dihedral angles ∼ 55°), and the **βC** compound has a planar PDI–BDT–PDI structure (dihedral angles ∼ 0°) (Fig. S28†). In both cases, the hole transition orbitals are localized on the electron-rich BDT units, and electron transition orbitals show more localization on the PDI acceptor unit(s) (Fig. 2). Also, the theoretical calculations reveal electron localization on only one PDI unit for the twisted **β** compound, but complete delocalization across all trimer units for the planar **βC** compound. This indicates minimal ground state interaction or excitonic coupling among the chromophores^35^ in **β** – hence its similar absorption peak (*λ*^max^) to that of the parent PDI monomer (Fig. 1). However, the spectral behavior of **βC** is indicative of a much stronger coupling among the PDIs, and with the BDT core.

**Table 1:** Absorption and fluorescence properties, and two-photon absorption cross sections for the trimers in chloroform

Comp.	*λ*_abs_/nm	*λ*_em_/nm	Δ*υ*^a^/cm^−1^	*ε* b/M^−1^cm^−1^	*ϕ*_F_/%	*δ*_TPA_/GM, *λ*_exc_ = 810 nm	*δ*_TPA_/GM, *λ*_exc_ = 875 nm
**β**	**526**, 630^(sh)^	790	3215	71 100	0.3	—c	11.5
**βC**	**510**, 605	613, 665^(sh)^	215	58 500	9	227	318

a Δ*υ* is the Stokes shift.

b At the bold wavelength.

c At *λ*_exc_ = 810 nm, strong interference from one photon excited fluorescence was observed due to the long wavelength tail of the **β** absorption spectrum (Fig. 1).

No concentration effect on the absorption spectral shape was observed in the range of concentrations employed in this investigation (see Fig. S1†), which are similar to, or lower than the ‘low concentration’/dilute limit employed in other literature studies about xSEF compounds in solution.^36^ Therefore, it is possible to rule out the occurrence of any intermolecular interactions due to aggregation which may affect our experimental results. Hence, the optical properties exhibited by the compounds investigated here are due to isolated molecules in solution.

The fluorescence quantum yield of the investigated compounds is low (0.3% and 9%, see Table 1) in comparison to that of the parent PDI monomer (88%).^37^ The fluorescence efficiency is 30 times lower in the case of compound **β** relative to **βC**. This behavior parallels the observed trend of the Stokes shift in the two trimers and agrees with the increased molecular rigidity of **βC** relative to **β**. More rigid molecular structures are indeed known to exhibit enhanced fluorescence capability.^33,38^ This result suggests that the excited state deactivation of these trimer compounds takes place mainly through non-radiative pathways – possibly triplet production/decay, in competition with the fluorescence decay pathway. This non-radiative deactivation is more efficient for the twisted **β** compound, whose fluorescence quantum yield is almost negligible.

Previous studies have observed the relationship between molecular planarity and two-photon absorption.^34,39,40^ Here, the two-photon absorption cross section (*δ*_TPA_) is enhanced by over one order of magnitude for the planar **βC** (*ca.* 300 GM) relative to the twisted **β** compound (*ca.* 10 GM). The increased two-photon absorption cross section of the planar, rigid, fused ring connected molecule, as expected, indicate its higher intramolecular charge transfer character in the excited state relative to the twisted, flexible, single bond bridged analogue. The degree of charge transfer for the **β** and **βC** excited state was further analyzed in detail with the help of quantum chemical simulations. These compounds were divided into 5 subunits/moieties, considering their acceptor(1)–donor(2)–acceptor(3)–donor(4)–acceptor(5) structure where the acceptors and the donors are the PDI the BDT fragments, respectively. The NTOs computed on the S_1_ geometry which describe the S_1_→S_0_ transition are shown in Fig. S31,† and the amount of charge transferred during emission is reported in Table S1.† The charge transfer degree for the excited state of **βC** (0.80 e^−^) is indeed higher than that of **β** (0.74 e^−^).

### Femtosecond transient absorption

**Fig. 3:**
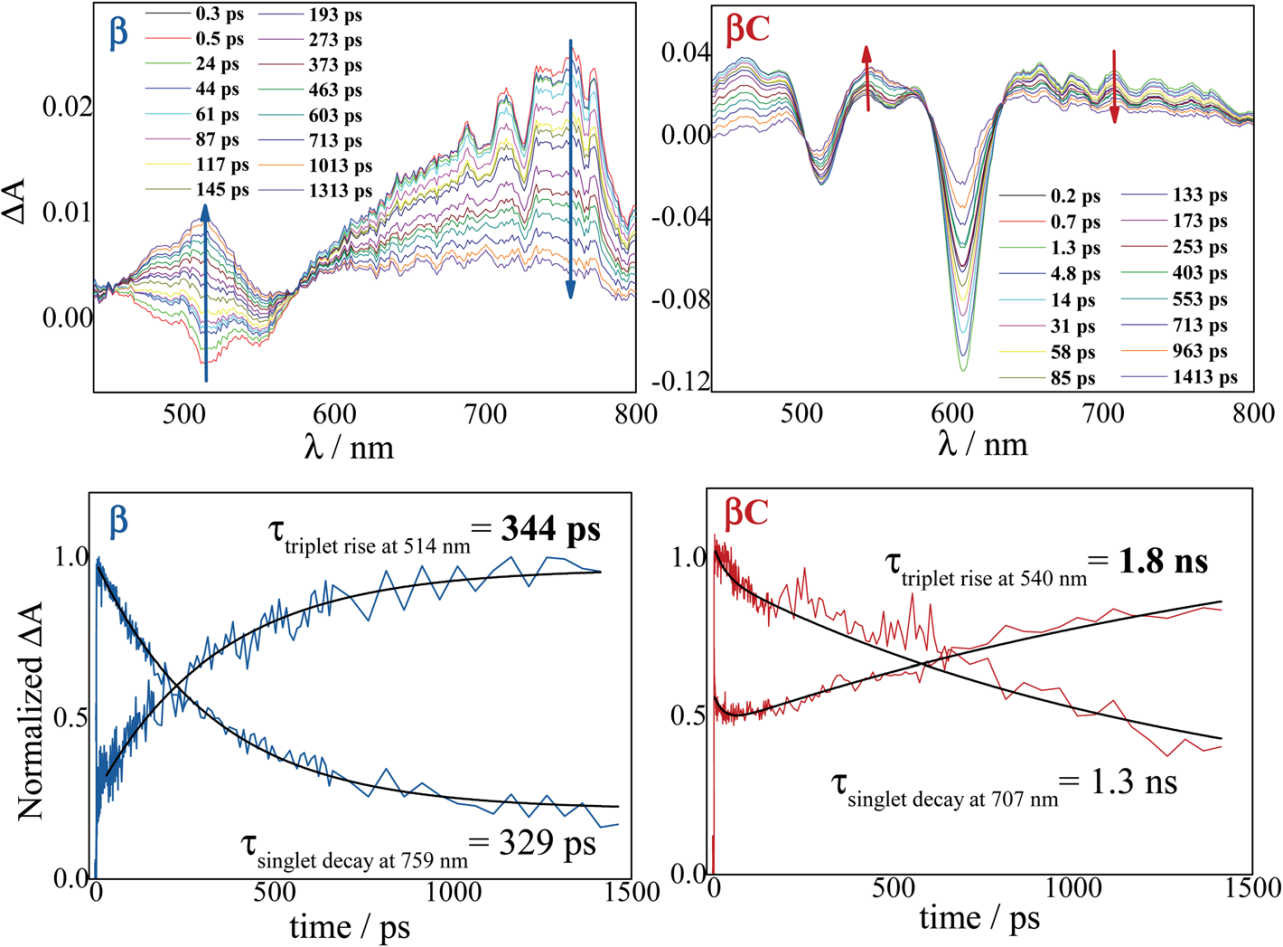
Time-resolved spectra obtained by femtosecond TA for the trimers in chloroform. Singlet decay and triplet rise kinetics for the trimers in chloroform.

The excited state dynamics was investigated by femtosecond transient absorption. The time resolved spectra (Fig. 3 & S3†) show positive excited state absorption (ESA) and negative Ground State Bleaching (GSB) signals. The ESA at 740 nm has been previously associated with the PDI anion, whereas signals between 550 and 600 nm have been assigned to the PDI cation.^41–47^ The transient spectra of the investigated trimers at short delays following photoexcitation suggest the occurrence of intramolecular charge transfer (ICT), and no significant spectral shift was observed. It is possible that a singlet excited state with ICT character is formed very fast (within solvation).^47^ At longer delays this signal decays, resulting in the simultaneous formation of an ESA at 514 nm and 543 nm for **β** and **βC**, respectively. The kinetics at these wavelengths exhibit a rise (Fig. 3). This rise occurs very fast for the twisted **β** compound (344 ps), but clearly slower for the rigid-bridged **βC** compound (1800 ps). Global analysis, *via* singular value decomposition (SVD), of the transient absorption data revealed the presence of four exponential components (Fig. S3B & Table S2†). The first two fast components can be associated to solvation and vibrational cooling/structural relaxation. The third component, assigned to the relaxed S_1_, shows a lifetime of 320 ps for the **β** compound and 1300 ps for the **βC** compound. The fourth component represents the rest species formed upon S_1_ decay and peaked around 510–550 nm. This long-lived species are triplets, as demonstrated by their spectral similarity to the species detected by nanosecond transient absorption (see next section). Therefore, the ultrafast absorption measurements allow us to follow the triplet formation dynamics in these molecules. The triplet formation occurs fast for **β** (∼340 ps) and much slower for **βC** pointing to a different mechanisms for triplet production in the two molecules – SEF for **β** and ISC for **βC**, respectively. Additionally, triplet formation takes place slower in a less polar solvent relative to chloroform (*e.g.* for **β** in toluene triplet rise occurs in *ca.* 690 ps).

Triplet quantum yields were also computed following the SVD analysis of the femtosecond transient absorption results^48–51^ (see ESI† for details on the procedure). Quantitatively related singlet and triplet ESA spectra were obtained by matching their GSB and then the temporal population dynamics of these states were determined. The population data (Fig. S8 & S13†) indicate a triplet quantum yield of 189% for **β** and higher than 46% for **βC**. Although this analysis contains some approximations, our result thus show that triplet production takes place *via* iSEF for the twisted **β** compound (*φ*_T_ close to 200%) and *via* conventional ISC for the planar/rigid-bridged **βC** system (*φ*_T_ ≪ 100%).

### Nanosecond transient absorption

**Fig. 4:**
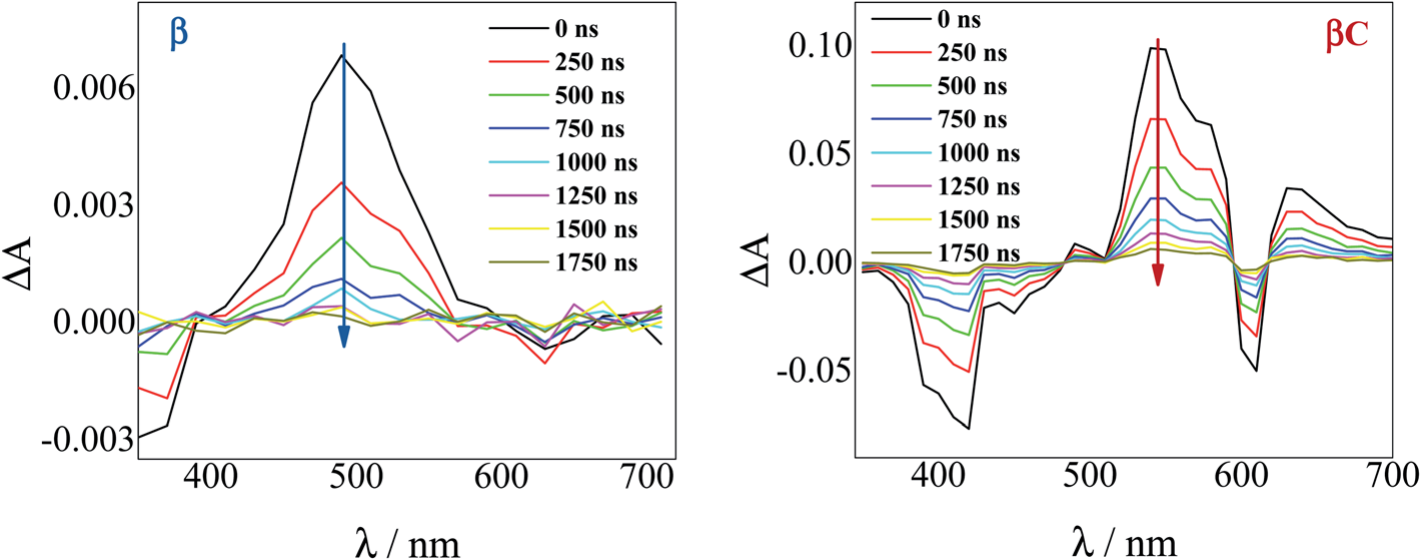
Time-resolved spectra obtained by nanosecond TA measurements for the trimers in air-equilibrated chloroform upon 415 nm laser excitation.

**Fig. 5:**
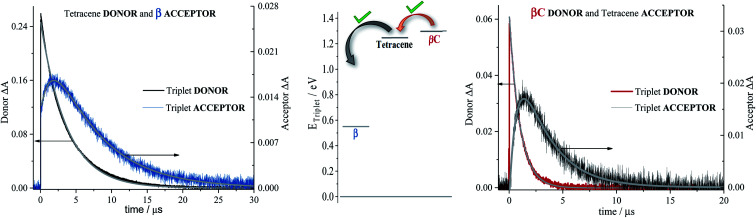
Decay and rise dynamics of trimers (extreme left for **β**; and extreme right for **βC**) in cyclohexane obtained by nanosecond TA for triplet sensitization measurements. Middle graph shows the triplet energy for samples and sensitizers.

To investigate the long-lived excited state dynamics, nanosecond transient absorption measurements were carried out (Fig. 4). No pump wavelength dependence was observed (see Fig. S14†). The transient spectra show negative signals due to GSB, and a positive ESA peak centered at 490 nm and 540 nm for **β** and **βC**, respectively. It is worthy to note that signals of triplet absorption have been reported for other PDI derivatives between 500 and 600 nm.^18,37,52,53^ This signal can be quenched by oxygen (either *via* energy transfer or electron transfer).^54^ The transient lifetimes change from hundreds of nanoseconds in air equilibrated solution to tens of microseconds in deaerated/nitrogen purged solution (Fig. S15† & Table 2). Quenching by molecular oxygen thus occurs at an almost diffusional rate (1.2 × 10^10^ M^−1^ s^−1^ in chloroform). Also, these transient species can be sensitized by higher-triplet energy donors, or are able to sensitize lower-triplet energy acceptors, such as tetracene as shown in Fig. 5. These results allow us to undoubtedly assign these long-lived transients revealed by nanosecond transient absorption experiments to the T_*n*_ ← T_1_ transition of the trimers. As shown in Table 2, for **β**, the triplets produced upon photoexcitation decay much faster *i.e.* shorter lifetimes (6.0 μs), in comparison to the triplets produced in **βC** (40 μs). This is an evidence leaning to a SEF-induced mechanism of triplet production in the twisted **β**. It has indeed been observed in many SEF literature studies^17,55–57^ that a molecule hosting two triplet excitons usually exhibits a much faster triplet decay than one hosting a single triplet, due to the increased probability of triplet–triplet annihilation.

**Table 2:** Triplet properties for the trimers in chloroform from nanosecond transient experiments

Comp.	*λ*_T_/nm	*τ*_T,air_ a/μs	*τ*_T,N_2__ b/μs	*k*_ox_/M^−1^ s^−1^	*ϕ*_T_ *ε*_T_/M^−1^ cm^−1^	*ε*_T_ c/M^−1^ cm^−1^	*ϕ*_T_
**β**	490	0.39	6.0^d^	1.0 × 10^9^	2785	1637	1.70
**βC**	540	0.50	40	0.8 × 10^9^	7460	52 800	0.16

a In air-saturated chloroform.

b In N_2_-saturated chloroform.

c In cyclohexane.

d ∼7 μs lifetime was obtained by triplet sensitization in cyclohexane, see Fig. S20.

Sensitization experiments were performed *via* nanosecond transient absorption measurements. These experiments give important information about the triplet energy of the compounds (see Fig. 5). Tetracene (*E*_T_ = 1.27 eV) was successfully employed as an energy donor to sensitize the triplet of **β**, but relatively acted as a triplet energy acceptor to **βC**. This result proves that the triplet energy of the twisted **β** trimer is significantly lower than the triplet energy of the planar **βC** trimer. Our experimental results thus support the feasibility of SEF in the **β** compound which requires a low triplet energy for the SEF energetic condition to be fulfilled (*E*_T_ = 0.55 eV; obtained from theoretical calculations). The sensitization experiments also allowed for the accurate evaluation of triplet extinction coefficients (see Table 2), useful for evaluating the singlet → triplet quantum yield. A very detailed step-by-step triplet extinction calculation for the two compounds is given in the ESI.† A lower extinction coefficient was observed for the twisted relative to the planar, rigid system. These experiments, together with the relative actinometry measurements described in the ESI,† allowed for the accurate computation of the singlet → triplet quantum yields. A triplet quantum yield of 16% was obtained for the planar **βC** compound, suggesting that conventional intersystem crossing occurs in this chromophore. Triplet yield significantly higher than 100% was obtained in the case of the twisted **β** compound (*ϕ*_T_ = 170%), thus suggesting that iSEF indeed takes place in this molecule.

### Two-color transmission measurements of triplet yield

**Fig. 6:**
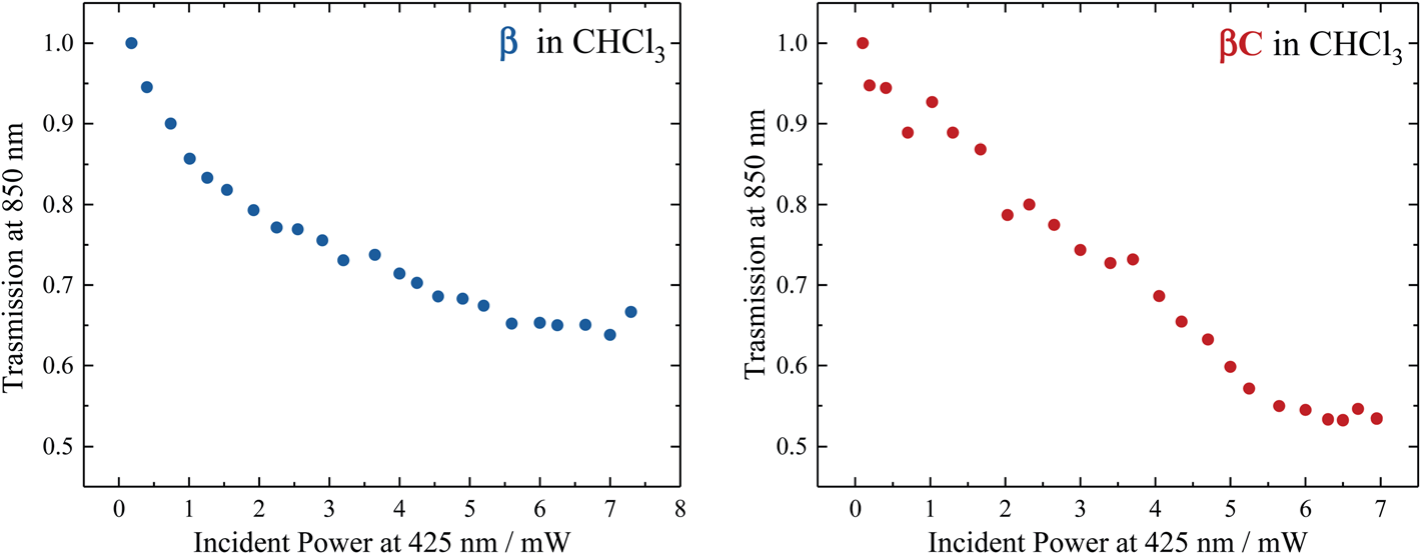
Transmission of **β** (left) and **βC** (right) in CHCl_3_ for the probe light at 850 nm as a function of the pump power at 425 nm.

To selectively probe the ESA without contribution from the GSB, the trimer compounds were investigated using two-color transmission spectroscopy.^28^ This was performed by probing the samples at 850 nm, where linear absorption is negligible, under excitation with femtosecond pulses at 425 nm. Attenuation of the probe beam was observed for both samples: 29.7% attenuation for **β** at OD = 0.116 and 34.6% attenuation for **βC** at OD = 0.885, both under an average pump power of 4.25 mW (Fig. 6). This demonstrates the accumulation of triplets upon irradiation of the trimers, observed to be more in the case of **β**. Indeed, the theoretical calculations predicted significant absorption by T_1_ species around 850 nm: transition T_1_ → T_8_ at 898 nm and T_1_ → T_10_ at 826 nm for **β**; transition T_1_ → T_16_ at 849 nm for **βC**.

Analysis of the obtained results was carried out in order to obtain an estimation of the triplet quantum yield for the two compounds. See the ESI† for the detailed calculation performed according to a procedure described in ref. 25. It entails computing the triplet number density using the 850 nm probe beam attenuation (as shown in Fig. 6) and the singlet excitation number density from the ground state OD and 425 nm pump beam parameters. The triplet extinction coefficients at 850 nm used for this calculation were obtained *via* nanosecond transient absorption measurements, by taking the ratio of the Δ*A* signal at 850 nm with that at the triplet peak for which the extinction coefficient is known (see Table 2, Fig. S24 and S25†). This was accurately done for **β** which had a distinct triplet ESA signal ∼850 nm (see Fig. S23†). However, for **βC** the triplet ESA signal was convoluted with the phosphorescence around 850 nm (see Fig. S23†). Therefore, the same ratio of the Δ*A* signal between the triplet peak and that at 850 nm for **β** was assumed for the **βC** molecule. From the calculation, the triplet number density was evaluated to be 2.32 × 10^10^ cm^−3^ for **β** and 8.61 × 10^8^ cm^−3^ for **βC**; the singlet number density 1.24 × 10^10^ cm^−3^ for **β** and 3.14 × 10^11^ cm^−3^ for **βC**. Therefore, a much higher triplet quantum yield was indeed obtained for the twisted **β** trimer (∼187%) relative to the planar **βC** (∼0.3%). It has to be noted that for the case of the **βC** compound, the estimated triplet yield value is not accurate because of the observed phosphorescence interference at 850 nm. This analysis contains some approximations, however the result obtained for the **β** trimer is consistent with the triplet quantum yield accurately measured by the nanosecond transient absorption sensitization experiments. This once again confirms that the **β** compound thus undergo singlet exciton fission in solution, owing to its triplet yield also obtained by two-color transmission measurements to be ≫100%.

### Time resolved fluorescence

Time resolved fluorescence measurements, both with femtosecond and nanosecond time resolution, have been extremely valuable in providing information about the rate constants of the ultrafast intramolecular charge transfer process and about the decay of the double triplet species, respectively. Fluorescence kinetics were acquired by femtosecond fluorescence up conversion (FUC) (Fig. S26 & S27†). Their fitting revealed the presence of exponential components (Table S5†), whose lifetimes agree with those obtained *via* femtosecond transient absorption measurements. The much smaller time window of the FUC allows for a more accurate evaluation of the lifetime of the ultrafast components, as 1.0 ps for **β** and 0.2 ps for **βC**. Our FUC results show that the ICT is indeed faster in the rigid relative to the twisted trimer. Also, that SEF could be a CT-mediated process in the twisted trimer.^10,22^ However, when ICT is extremely fast it becomes competitive with SEF, as observed in the planar trimer.^50^

**Fig. 7:**
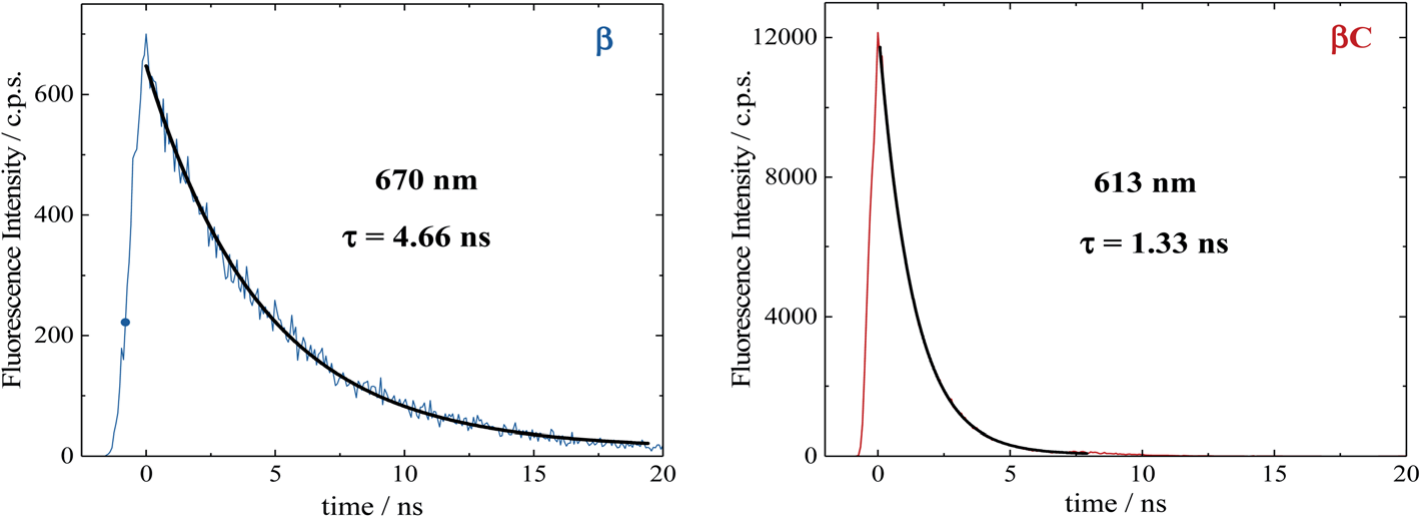
Fluorescence kinetics obtained by nanosecond TCSPC in air equilibrated chloroform.

Fluorescence kinetics were also acquired by single photon counting (SPC) with nanosecond resolution (Fig. 7). For the rigid **βC**, these experiments revealed a lifetime of 1.33 ns, in agreement with the femtosecond TA measurement – 1.3 ns (Fig. 6 & Table S4†). For **β**, the SPC fluorescence decay is surprisingly slower, and the fitting revealed a lifetime of 4.66 ns. This fluorescent component exhibits a lifetime quite different from that estimated by the femtosecond transient absorption for S_1_ – 320 ps, and by the high resolution FUC. Therefore, the 4.66 ns component may be due to a precursor of T_1_, possibly a double triplet excited state ^1^(TT)^*^. This component could be either a result of direct ^1^(TT)^*^ emission,^58–60^ or delayed S_1_ fluorescence from the ^1^(TT)^*^ state.^1,19,23,53^ Time resolved emission and/or temperature-dependent spectra obtained by photoluminescence measurements with broadband detection would be required to identify the specific mechanism of the double triplet emission. A 4.66 ns lifetime is not unusual for double triplet states as observed in the literature for perylene diimide chromophores. Wasielewski *et al.*^18^ reported a lifetime of 2.8 ns for polycrystalline thin films of slip stacked PDI, attributed to a small amount of delayed fluorescence resulting from triplet–triplet annihilation. Sean Roberts *et al.*^53^ also reported lifetime of ∼10 ns associated to the non-radiative decay of the triplet (or double triplet) excitons to the ground state.

### Quantum chemical simulations: intersystem crossing and singlet fission

Quantum chemical simulations were performed to give an insight into triplet formation mechanisms. Triplet formation *via* ISC was predicted to be much faster for the planar **βC** than for its twisted analogue due to large cancellation between Δ*E*_ST_ and reorganization energies for **β** (*i.e.* 8.44 × 10^5^ s^−1^/1185 ns for **β** and 1.35 × 10^7^ s^−1^/74.1 ns for **βC**, see ESI†). However, experimentally, the long-lived triplet species appear much faster (340 ps) for the twisted **β**. This implies the existence of another pathway of triplet generation: iSEF. Similar timescales of triplet formation *via* SEF in other PDI derivatives have been reported.^18,53^ TD-DFT was used to illustrate the SEF relevant energetics and to check if the energetic requirement or *thermodynamic feasibility*, *E*(S_1_) ≥ 2 × *E*(T_1_), is met. Energies of the relaxed S_1_ state were predicted to be 1.30 eV and 1.89 eV for **β** and **βC**, respectively (Fig. S30†). T_1_ state energies at its minimum structures (Fig. S29†) were 0.55 eV and 1.24 eV for **β** and **βC**, respectively. These energetics indicates that SEF in the twisted **β** is thermodynamically favorable by 0.20 eV (2 × 0.55 − 1.30 = −0.20), but not in the planar **βC** for which it is energetically uphill by 0.59 eV (2 × 1.24 − 1.89 = 0.59).

**Fig. 8:**
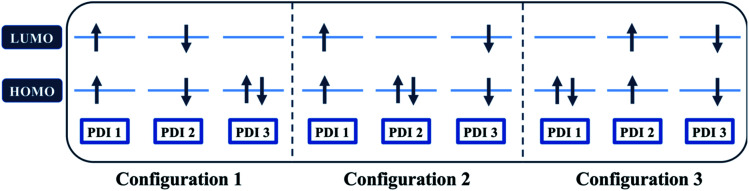
Possible electronic configurations of active space orbitals for both **β** and **βC** compounds.

**Fig. 9:**
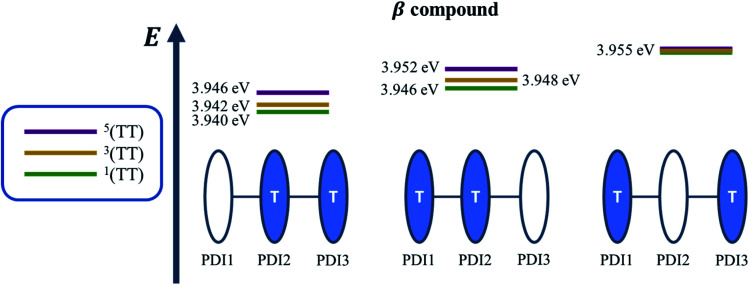
Energy level diagram illustrating the relative energies of all the double-triplet states found in **β** compound, with the colored PDI chromophores indicating where the triplet excitons are located.

**Fig. 10:**
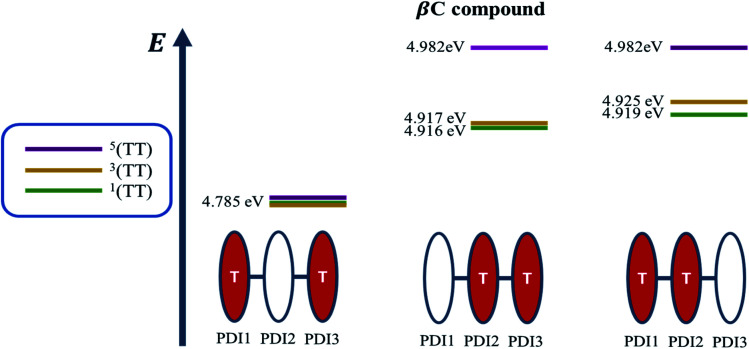
Energy level diagram illustrating the relative energies of all the double-triplet states found in **βC** compound, with the colored PDI chromophores indicating where the triplet excitons are located.

A recent perspective pointed out that the accessibility of double triplet state of singlet character (^1^TT), that is the *kinetic feasibility*, is more instrumental to judge the potentiality of SEF taking place than just the simple singlet–triplet energy gap.^61^ Though TD-DFT calculations were conducted to obtain the energetics of singly excited states, it is clearly stated by theorists that TD-DFT is not an ideal method for calculation of the multi-excitonic (ME) states, *i.e.*, double-triplet states. The RAS-SF method, on the other hand, has shown to be capable of correctly describing the characters of multi-excitonic states and providing a more in-depth picture of the interactions between the locally excited singlet and multi-exciton states. Importantly for trimers like **β** and **βC**, RAS-SF is also able to compute all possible multi-exciton states, obtaining their spatial as well as their spin components (Fig. 8, 9 and 10). RAS-SF can provide the relative energies of all the double–triplet states, and identify behavior discrimination between these states in each trimer. However, RAS-SF overestimates the excitation energies because of an incomplete account of dynamic correlation.^21,62^ Even though the absolute energies are not accurate (and this explains the poor agreement with the DFT energies), the trends and the relative energy values can still be discussed. The TT states from the RAS-SF trimer models are qualitatively described in Fig. 8. Detailed descriptions of the frontier orbitals for these states can be found in Fig. S34 and S35.†

Krylov has shown that it is not uncommon for perylene diimide compounds to have their lowest ^1^TT state above their lowest excited singlet exciton state.^21^ In **β** and **βC**, the lowest ^1^TT state is placed 0.49 eV and 1.10 eV above their respective S_1_ state (Table S7†). This ^1^TT state becomes nearly inaccessible in the **βC** compound due to an additional energy of more than twice that of the **β** compound (0.61 eV), required to reach the multiexcitonic state from the S_1_ state. As shown in Fig. 9, the multi-excitonic state with lowest excitation energy for the **β** compound is configuration 3. More specifically, the RAS-SF calculation shows that excitons in the lowest multi-exciton state are localized on *adjacent* PDI units. The two higher energy configurations (configurations 1 and 2) are within 0.02 eV of configuration 3, indicating that all TT states of **β** compound are easily energetically accessible. In the case of the **βC** compound, the lowest multi-exciton state is configuration 2 of Fig. 8, where the triplet excitons reside on the left-most and right-most PDI units. The energy of this state is about 0.2 eV under that of the two other configurations (one order of magnitude higher in comparison to **β**), suggesting a nontrivial difference in energy to access configurations 1 and 3.

A photo-excited singlet state S_1_ can evolve into a triplet-paired state^1^(TT) in singlet fission chromophores *via* state crossings when the S_1_ and TT states are close in energy.^63^ Throughout this non-adiabatic transition, it is true in some cases that the singlet exciton of S_1_ state resides over several adjacent chromophores. This phenomenon promotes the ^1^(TT) formation and thus increases the SF efficiency.^61^ Only the couplings of the singly excited state S_1_ with the ME state on adjacent chromophores will be playing significant roles throughout this non-adiabatic transition. In this particular case of **β** compound, as suggested by the electronic configuration of its lowest TT state (Fig. 9), the S_1_ exciton is located on two adjacent units, PDI2 and 3. This spatial characteristic explains why the TT formation is promoted in this **β** compound. Nevertheless, this S_1_ exciton is spread out on the two isolated units in **βC**. As discussed above, since the S_1_ excitons are less likely to reside across PDI1 and PDI3, it is therefore not surprising to conclude that forming the TT state is more difficult in **βC** than it is in **β** compound.

Additionally, performing analysis on energy differences between ^1^TT state and quintet state energies allows us to judge the *feasibility of separation of the double triplet into two independent triplets*. This energy difference, sometimes called inter-triplet interaction energy, is also known as the energy penalty for separating two triplets. This inter-triplet interaction energy accounts for the unmixing of charge transfer contributions in the singlet TT state by comparing the ^1^TT state to the corresponding quintet state, which always is a pure diabatic TT state.^21^ For the twisted **β** compound, the ^1^TT states are nearly degenerate with their corresponding quintet states, giving inter-triplet interaction energies of 0.006 eV for the two lower multi-exciton states (Fig. 9). This result suggests that the interaction between two triplets in **β** is quite small, and two entangled triplets can thus easily be separated into two independent triplets. In **βC** compound, however, the inter-triplet interaction energies increase by an order of magnitude, up to 0.066 eV, for the two higher multi-exciton states (Fig. 10). This result entails that the formation of double triplets and the subsequent separation of entangled triplets require much less energy for **β** than for **βC**. Another notable fact is that the lowest ME state in the planar **βC** compound, which is the same case as the highest ME state in the twisted **β** compound, has no other but only the pure ME contributions toward the states. This fact also makes sense since the two triplet excitons are located on the two isolated chromophores so that it is apparently harder for charge transfer contribution to play a role in this particular situation.

Overall, quantum chemical simulations support that iSEF is the dominant pathway to generate *independent* triplets only in **β** but not in **βC**, based on the thermodynamic viability (Δ*E*_S–2×T_), kinetic accessibility, and feasibility of separation of the double triplets.

## Discussion

In the literature, it has been proposed for multichromophoric systems that using a rotatable linker is crucial in obtaining iSEF.^15–17^ Those studies, however, do not report the direct comparison between rigid-bridged and flexible-bridged units of the same chromophore in order to isolate the effect of the π-bridge. In this work, we point out the key differences between planar (rigid-bridged) and twisted (flexible-bridged) systems as they relate to the efficiency and rate of triplet production upon singlet photoexcitation. The systems investigated are newly synthesized oligomeric PDI trimers. Typically, the fluorescence quantum yield of PDI monomers is around 90%, indicating that the radiative decay pathway is the most preferred. However, for these PDI trimers, especially for the flexible-bridged **β** trimer, the fluorescence efficiency is found to be very low suggesting a prevalent non-radiative deactivation – triplet production.

**Chart 2:**
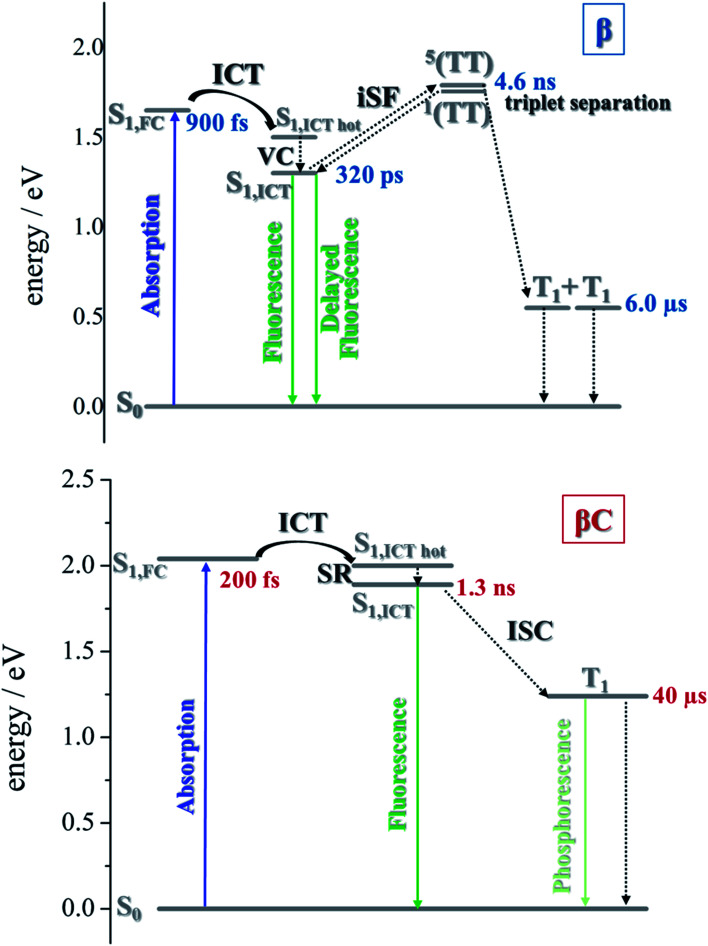
Sketch of the proposed excited state deactivations, based on the excited state energies predicted *via* quantum simulations and the excited state dynamics observed *via* time resolved spectroscopic experiments.

For the planar **βC** trimer, we obtain a triplet yield of 16% *via* triplet sensitization experiments employing a nanosecond transient absorption technique. The femtosecond transient absorption results show ultrafast intramolecular charge transfer and slow triplet formation occurring in few nanoseconds for this molecule. This rate agrees with the intersystem crossing rate predicted by quantum chemical simulations. Our experimental and computational results thus conclude that triplet production for the rigid **βC** trimer proceeds *via* regular intersystem crossing (Chart 2). Conversely, in the case of the flexible-bridged **β** trimer we clearly show that the mechanism of triplet production is different and involves iSEF (Chart 2), based on the following evidences. (i) Triplet yield ≫ 100%, obtained *via* triplet sensitization as well as two-color transmission experiments. (ii) A fast triplet formation (∼340 ps) observed *via* femtosecond transient absorption measurements. (iii) Distinct triplet species detected *via* transient absorption experiments – the correlated triplet pair and the independent triplets with lifetime of 6 μs.^64–66^ (iv) A decay lifetime different from that of the S_1_ species (4.7 ns), attributed to the double triplet species. (v) Thermodynamic viability – *E*(S_1_) ≥ 2 × *E*(T_1_). (vi) Kinetic feasibility allowed by the energetic accessibility of the double triplet state from the S_1_ state – the rate is given as: *r* ≈ e^−const.(*E*S_1_−*E*^1^TT)^.^21^ Our findings thus suggest that iSEF takes place in the flexible-bridged **β** trimer. *As an important result of our study, we demonstrate that the rotational flexibility of the linker, as in the **β** trimer compound, is necessary to activate multiexciton triplet generation in multichromophoric PDIs.* Our findings constitute a significant progress in structure–property relationships and may drive future design of extremely efficient iSEF materials.

To gain a deeper insight into the thermodynamics of multiexciton generation, we obtained all the relevant enthalpic and entropic quantities for the trimers following an approach proposed by Krylov *et al.*^21,67^ Multiexciton generation was considered to occur in two steps: a first step proceeding from the excited singlet to the double triplet state (from S_1_ to ^1^TT) and a second step leading to triplet separation (from ^1^TT to T_1_). The total enthalpy and entropy change were evaluated as the sum of the changes observed in the two steps. Detailed results are reported in Table S8.† The SEF process is indeed exothermic for the **β** twisted compound (Δ*H*_TOT_ = −0.20 eV) and endothermic for the **βC** planar trimer (Δ*H*_TOT_ = +0.59 eV). In the literature about iSEF in covalently linked dimers, only enthalpy has been considered in describing their thermodynamics. This is because covalently linked dimers can accommodate just one correlated triplet pair, hence no entropic contribution. However, for trimeric structures the role of entropy should be considered. In a covalently linked trimer, like those investigated here, the double triplet has three possible accommodations on two of its three PDIs; and in particular two equivalent accommodations on each pair of adjacent PDIs owing to molecular symmetry. In principle, the trimeric structure allows entropic gain to play a significant role in the SEF thermodynamics, as literature studies have reported for solid state films^8,67^ and very recently for oligomers.^56^ The quantum chemical simulations indeed showed the presence of three multiexcitonic states. For **β**, the two ^1^TT accommodated on adjacent PDIs of the structure, as well as that on the left-most and right-most PDI units, are energetically accessible from S_1_. The *high triplet yield and fast iSEF rate observed in***β***is not only due to the enthalpic viability but also to the entropic gain (TΔS*_*TOT*_*= +0.028 eV) allowed by its trimeric structure*. This leads to a negative total change in Gibbs energy for **β** (Δ*G*_TOT_ = −0.228 eV), confirming the iSEF thermodynamic feasibility. On the other hand, for **βC**, the lowest energy ^1^TT state is the one for which the triplet excitons reside on the left-most and right-most PDI units whereas the two ^1^TT states allowing for an entropic gain are enthalpically inaccessible from S_1_. This leads to no entropic contribution, and since the total change in Gibbs energy is positive (Δ*G*_TOT_ = +0.59 eV), iSEF is not thermodynamically feasible for the planar **βC** trimer.

While strong coupling among the PDI chromophores can be inferred for the planar **βC** trimer, for the twisted **β** compound, weak coupling is observed because the singlet electron NTOs are localized on a single PDI unit. We suggest that the poor electronic communication among the PDI chromophores caused by the rotatable linker is crucial in permitting efficient iSEF. Our results demonstrate that the single-bond connections capable of weakening the coupling between the chromophores favor high iSEF yield. The fused ring connections induce strong coupling among the PDI units, as the singlet electron NTOs are delocalized over the planar trimer structure, and inhibit SEF completely. This is due to competition with other ultrafast processes in the planar system, such as intramolecular charge transfer.^26^ The two-photon absorption (TPA) cross section – a measure of intramolecular charge transfer character – is indeed enhanced by over one order of magnitude for the planar **βC** (*ca.* 300 GM) with respect to the twisted **β** compound (*ca.* 10 GM). Quantum chemical simulations confirm the higher degree of charge transfer in the excited state for **βC** (0.80 e^−^) relative to **β** (0.74 e^−^) by dividing their structure into 5 subunits of PDI acceptor and BDT donor fragments. Weak coupling among the PDI units is crucial not only for multiexciton generation but also for triplet separation. The rate of triplet separation is indeed given as: *r* ≈ e^−const.(*E*^5^TT−*E*^1^TT)^ by Krylov *et al.*^21^ For the twisted **β** compound, the ^1^TT states are nearly degenerate with their corresponding ^5^TT states, giving inter-triplet interaction energies (*E*^5^TT − *E*^1^TT) as 0.006 eV. This result suggests that the interaction between two triplets in **β** is indeed small, and two entangled triplets can thus easily be separated into two independent triplets. However, in **βC**, the inter-triplet interaction energies increase by one order of magnitude, up to 0.066 eV. For the **β** trimer, the long-lived independent triplets are experimentally observed following their separation, and each of them is localized on a single PDI unit, as shown by the theoretical calculations. Compared to other SEF rylene derivatives reported in the literature,^19,50,53^ the **β** trimer shows a much longer triplet lifetime. This is highly beneficial for its use in solar energy conversion devices, allowing efficient extraction of multiple charge carriers per incident photon.^68^

## Conclusions

Here, we report a comparative study between rigid-bridged (planar) and flexible-bridged (twisted) perylene diimide trimer systems to highlight the role of the π-bridge linker in activating intramolecular singlet exciton fission. We show *via* time resolved spectroscopic measurements how a slight structural variation of the π-bridge of multichromophoric perylene diimide (PDI) systems strongly affects the triplet yield and triplet formation rate. Triplet formation proceeds *via* conventional intersystem crossing for the planar trimer as evidenced by its triplet yield of 16% and triplet production in the nanosecond timescale. On the other hand, we find clear evidence of highly efficient (170%) and fast (few hundred picoseconds) intramolecular singlet exciton fission taking place in the twisted trimer. A fused ring connection induces strong coupling among the PDI units as in the planar system and this inhibits singlet exciton fission completely due to a strong competition with other ultrafast processes, such as intramolecular charge transfer. Our results demonstrate that a rotatable π-bridge, capable of lowering the coupling between the chromophores, is necessary to activate intramolecular singlet exciton fission in multichromophoric systems. The quantum chemical simulations prove that the entropic gain allowed by multiple possibilities of accommodating the correlated triplet pair on adjacent PDIs in the twisted trimer is a crucial determinant for multiexciton triplet generation. Successive multiexciton triplet separation occurs in the flexible-bridged PDI due to weak coupling among the units, and degenerate double triplet and quintet states.

## Conflicts of interest

All authors have given approval to the final version of the manuscript and declare no competing financial interest.

## Supplementary Material

SC-011-D0SC03271A-s001
