# Supplementary material for: Activating intramolecular singlet exciton fission by altering π-bridge flexibility in perylene diimide trimers for organic solar cells
Source: Chem Sci. 2020 Aug 7;11(33):8757–70. doi: 10.1039/d0sc03271a (PMC8163386; doi:10.1039/d0sc03271a)
Supplement: SC-011-D0SC03271A-s001 [file SC-011-D0SC03271A-s001.pdf]

Electronic Supplementary Material (ESI) for Chemical Science.
This journal is © The Royal Society of Chemistry 2020

SUPPORTING INFORMATION

Activating Intramolecular Singlet Exciton Fission by Altering π−Bridge Flexibility in Perylene diimide Trimers for Organic Solar Cells

Benedetta Carlotti,[1,2,Ψ] Ifeanyi K. Madu,[1,Ψ] Hyungjun Kim, [1,3] Zhengxu. Cai,[4] Hanjie Jiang,[1] Angelar K. Muthike,[1] Luping Yu,[4] Paul M. Zimmerman, [1] Theodore Goodson III [1,]*

[1] Department of Chemistry, University of Michigan, Ann Arbor, MI 48109, United States

[2] Department of Chemistry Biology and Biotechnology, University of Perugia, via Elce di Sotto n.8, 06123 Perugia, Italy

[3] Department of Chemistry, Incheon National University, Incheon, 22012, Republic of Korea

[4] Department of Chemistry and The James Franck Institute, The University of Chicago, 929 East 57th Street, Chicago IL 60637, United States

Ψ B. Carlotti and I. K. Madu contributed equally to this work as first authors.

* To whom correspondence should be addressed. Email: tgoodson@umich.edu

Table of Contents

1. Experimental Section **S3**

2. Steady–State and Two–Photon Absorption Measurements **S7**

3. Femtosecond Transient Absorption (fsTA) **S8**

4. Triplet Yield Calculation from fsTA ....

β Trimer S8

βC Trimer S11

5. Nanosecond Transient Absorption (nsTA) **S14**

6. Triplet Sensitization Experiments by nsTA ....

Triplet extinction coefficient and Yield of β S16

Triplet extinction coefficient and Yield of βC S18

7. Two–Color Transmission Measurement of Triplet Species to compute Triplet Yield **S20**

8. Fluorescence Up–Conversion (FUC) **S24**

9. Quantum Chemical Simulations **S25**

10. ^{1}H and ^{13}C NMR of the compounds **S32**

1. Experimental Section

Materials, Synthesis and Characterization. The trimer structures were synthesized according to the synthetic route described in Chart 1. All of the chemicals were purchased from Aldrich. All reagents purchased commercially were used without further purification except for toluene and tetrahydrofuran (THF), which were dried over sodium/benzophenone. ^{1}H NMR and ^{13}C NMR spectra were recorded on a Bruker DRX–500 spectrometer with tetramethylsilane as an internal reference. High Resolution MALDI–TOF spectra were recorded on Bruker Solarix 9.4T. Compounds **β** and **βC** were synthesized according to the procedures developed in our lab.[1]

*Synthesis of Compound **β**.* To a round–bottom flask equipped with a condenser, **1** (311 mg, 0.37 mmol), **2** (170 mg, 0.41 mmol), **$Pd_2(dba)_3$** (34 mg, 0.04 mmol), and **$P(o\text{-}MePh)_3$** (45 mg, 0.14 mmol) were added. The system was evacuated and refilled with N_2 three times, then charged with toluene (50 mL). The reaction mixture was refluxed under N_2 for 8 h. Then, compound **3** (102 mg, 0.11 mmol), **$Pd_2(dba)_3$** (8.5 mg, 0.01 mmol), and **$P(o\text{-}MePh)_3$** (12 mg, 0.04 mmol) were added. The reaction mixture was refluxed under N_2 for another 8 h. After cooling to room temperature, the reaction mixture was concentrated under reduced pressure. The residue was purified by column chromatography with hexane and CH_2Cl_2 (1:1, v/v) as the eluent. Compound **β** was obtained as a red solid (292 mg, 25.4% yield). ^{1}H NMR ($CDCl_3$): δ 8.87 (m, 4H), 8.70-8.65 (m, 8H), 8.35 (s, 4H), 8.23 (m, 4H), 7.77 (s, 2H), 7.68 (s. 2H) 5.19 (m, 6H), 3.14 (s, 4H), 3.02 (s, 4H), 2.25 (m, 12H), 1.84 (m, 16H), 1.23 (m, 130H), 0.83 (m, 48H), 0.74 (m, 12H). ^{13}C NMR ($CDCl_3$): δ 144.4, 144.1, 139.6, 139.5, 137.9, 137.6, 134.2, 133.9, 133.9, 133.6, 130.1, 130.0, 129.9, 129.2, 129.1, 128.4, 128.1, 127.5, 123.7, 123.0, 122.6, 122.0, 54.7, 40.3, 38.4, 32.4, 31.8, 31.7, 29.3, 29.2, 28.9, 28.8, 27.0, 26.9, 23.0, 22.6, 22.6, 14.1, 14.1, 14.1, 14.0, 11.2, 11.1. MALDI-TOF: calcd. for [$C_{202}H_{256}N_6O_{12}S_3$], 3085.9, found, 3085.018.

*Synthesis of Compound **βC**.* A solution of $FeCl_3$ (800 mg, 4.9 mmol) in 2 mL nitromethane was added dropwise to a stirred solution of compound **β** (170 mg, 0.55 mmol) in 10 mL CH_2Cl_2. The reaction was stirred with Ar. After stirring for 10 h at room temperature, 1 mL methanol was added to the solution. The solvent was evaporated under reduced pressure, and the crude product was filtered with silica gel with a large amount of $CHCl_3$ to yield the solid product (158 mg, 93%).^{1}H NMR ($C_2D_2Cl_4$, ppm, 353 K): δ 10.54 (d, 2H), 10.39 (d, 2H), 9.99 (s, 2H), 9.93 (s, 2H), 9.35 (m, 4H), 9.08 (m, 4H), 5.48-5.21 (m, 6H), 4.13 (Br, 8H), 2.31-1.98 (br, 28H), 1.37-1.20 (m, 130H), 0.85-0.79 (m, 60H). ^{13}C NMR cannot be measured due to the aggregation issue. MALDI-TOF: calcd. for [$C_{202}H_{248}N_6O_{12}S_3$], 3077.8, found, 3077.9.

Steady−State Measurements. All of the measurements were performed at room temperature. Concentrations ranging from 1.6×10^{-6} to 1.6×10^{-4} M were used for the spectroscopic investigations. Absorption spectra were measured using an Agilent 8432 UV−visible absorption spectrophotometer. The emission spectrum measurements were performed with a Fluoromax−2 spectrofluorimeter. The fluorescence quantum yields of the samples were calculated using a known procedure[2,3] and Rhodamine B in ethanol (ϕ_F = 0.68)[4] was used as the standard.

Two−Photon Excited Fluorescence Measurements. Two−photon excited fluorescence measurements were performed using a Kapteyn Murnane (KM) mode−locked Ti:Sapphire laser tunable from 700 to 900 nm, and delivering 110 fs output pulses at a repetition rate of 80 MHz as described previously.[1,5,6] Emission scans were performed either at 820 or 875 nm excitation while scanning the emission in the 400−850 nm range, but the exact emission detection wavelength during the power dependence scan was selected by the emission wavelength that produced the highest number of counts. Input power from the laser was varied using a variable neutral density filter. Two−photon power−dependent fluorescence intensity was utilized to determine the two−photon

absorption cross section using the comparative method. Rhodamine B in ethanol was used as the standard (cross section 120 GM at 820 nm and 30 GM at 875 nm).[7]

Femtosecond Transient Absorption. An amplified laser (Spectra Physics Spitfire) with pulse duration of ~100 fs, repetition rate of 1 kHz, and power of 1 W was directed at a beam splitter to generate the pump (85%) and the probe beams (15%). The pump beam (~66 mJ per pulse) was generated from the second harmonic of the amplifier's output (~405 nm) using a BBO crystal in an optical parametric amplifier (Spectra Physics OPA-800CF) and was focused onto the sample cell (l = 2 mm) preceded by an optical chopper. The probe beam was passed through a computer−controlled delay line and focused onto a 2 mm sapphire plate to generate the white light continuum (Ultrafast Systems Inc.).[8,9] The white light was focused onto the sample and overlapped with the pump beam. The absorption difference (ΔA) of the signal was collected by a CCD detector (Ocean Optics). Data acquisition was performed with the software Helios by Ultrafast Systems Inc. The IRF was measured by the Raman scattering of water at 466 nm and is found to be 110 fs. Data analysis was performed with Surface Xplorer Pro and Glotaran softwares. All the experiments were performed in dilute solutions, at concentrations below 1×10^{-4} M.

Nanosecond Transient Absorption. The spectral properties and the lifetimes of long lived transient species were probed by transient absorption with nanosecond time resolution measurements.[10] These experiments were performed in dilute deaerated solutions, where photodegradation was checked by recording UV−vis absorption spectra before and after each experiment. All the experiments were performed in dilute solutions, at concentrations below 7×10^{-5} M. An LP980 (Edinburgh) spectrometer system, with a monochromator and PMT for signal detection (PMT−LP), was coupled with a Spectra Physics QuantaRay Nd:YAG nanosecond pulsed laser and a GWU Optical Parametric Oscillator (OPO) tunable for the excitation source. Flash lamps excite the ND:YAG rod in the laser head containing a polarizer, pockel cell, and ¼ wave plate, producing Q-switched 1064 nm light. Two-stage harmonic generation then produces high energy 355 nm light used to pump the OPO. The OPO produces excitation source from 206 nm to 2600 nm employing second harmonic generation and sum-frequency mixing nonlinear processes. For this investigation, 415 nm, 441 nm and 510 nm excitation wavelengths were used to pump the samples and a pulsed xenon lamp white light continuum was used to probe the absorption properties of the produced excited states. Relative actinometry measurement,[11,12] using an optically matched solution of Tetracene (ϕ_T = 0.62 and ε_T = 31200 M^{-1} cm^{-1} at λ_T of 465 nm)[13] in chlorobenzene as reference, was used to compute the product of the triplet yield and triplet−triplet extinction coefficient ($\phi_T \cdot \varepsilon_T$) of the samples. The same OD was maintained at 441 nm excitation λ for both reference and sample, hence generating equal concentration of singlets in both solutions. Then knowing that: $[singlet] = [triplet]/\phi_T$ and using Beer-Lambert's law: $[triplet] = \Delta A/l \cdot \varepsilon_T$ for both the sample and the reference, the product: $\phi_T \cdot \varepsilon_T$ of the sample can be computed. To compute the triplet yield (ϕ_T), triplet–triplet absorption coefficients (ε_T) were determined by triplet energy transfer measurements[14–16] to and from Tetracene in cyclohexane. Tetracene (E_T = 1.27 eV) was employed as a triplet energy donor for **β**, but as an acceptor for **βC**, giving a qualitative hint about their triplet energies i.e. $E_T < 1.27$ eV for **β**, but $E_T >$ 1.27 eV for **βC**. For computing ε_T successfully, in the donor + acceptor mixture, at the donor λ_T there has to be a decrease in the triplet lifetime (i.e. higher decay rate) in comparison to its lifetime with only the donor in solution. Also, at the acceptor λ_T, a triplet concentration rise has to be observed. These observations thus confirm triplet energy transfer from a donor to an acceptor. Using the decay rate of the donor only (k_D) and the acceptor (k_A) alongside the rate of energy transfer (k_{ET}), the ΔA_A and ΔA_D, the ε_T of donor or acceptor can be evaluated knowing the ε_T of the other.

Two–Color Transmission Measurements of Triplet Yield. Femtosecond two-color transmission measurements were carried out using a tunable Mai Tai laser system (Spectra Physics) giving 130 fs

pulses with a repetition rate of 80 MHz, tunable from 700 – 900 nm.[17] The light with wavelength of 850 nm plays the role of probe beam, and second harmonic generated (425 nm) light using a BBO crystal was used as the pump beam. To obtain the transmission profile, the excited state absorption at 850 nm was investigated with pump irradiation at 425 nm. The selected wavelength region is reasonable since there is negligible steady-state absorption at 850 nm for the investigated samples. A variable neutral density filter is placed to modulate the excitation power. The position of focusing lens is adjusted to place the focusing point on the sample. Blank chloroform solvent gives a reference line. A calibrated photodiode was used to measure the pump power. The transmitted power has been measured with a wide aperture power meter which is free of any thermal lensing effect.

Time−Resolved Fluorescence Measurements. Time−correlated single photon counting (TCSPC) technique, which has been described previously,[9] was used to study the long decay component of the investigated samples. The laser used for the TCSPC measurements was a home-built mode−locked Ti−sapphire laser with cavity dumper with repetition rate below 700 kHz. The output beam from the KM laser was at 800 nm wavelength, with a pulse duration of ca. 30 fs. The output beam was frequency−doubled using a nonlinear barium borate crystal to obtain a 400 nm beam. A polarizer was used to vary the power of the 400 nm beam that excites the sample. Focus on the sample cell (quartz cuvette, 0.4 cm path length) was ensured using a lens of focal length 11.5 cm. Collection of fluorescence was carried out in a direction perpendicular to the incident beam into a monochromator, and the output from the monochromator was coupled to a photomultiplier tube, which converted the photons into counts.

The femtosecond−resolved fluorescence experiments were performed using an ultrafast fluorescence Up−Conversion setup that had previously been described.[8,18–20] Mode−locked Ti−Sapphire femtosecond laser (Spectra Physics Tsunami) was used to generate 80 fs pulses at 800 nm wavelength with a repetition rate of 82 MHz. This mode−locked laser was pumped by a 532 nm continuous light output from another laser (Spectra Physics Millennia), which has a gain medium of neodymium−doped yttrium vanadate (Nd:YVO_4). A 400 nm excitation pulse was generated by a second harmonic β−barium borate crystal, and the residual 800 nm beam was made to pass through a computer−controlled motorized optical delay line. The polarization of the excitation beam was controlled by a Berek compensator. The power of the excitation beam varied between 33 to 36 mW. The fluorescence emitted by the sample was up−converted by a nonlinear crystal of β−barium borate by using the residual 800 nm beam, which had been delayed by the optical delay line with a gate step of 6.25 fs. By this procedure, the fluorescence can be measured temporally. The monochromator is used to select the wavelength of the up−converted beam of interest, and the selected beam is detected by a photomultiplier tube (R152P, Hamamatsu, Hamamatsu City, Japan). The photomultiplier tube converts the detected beam into photon counts, which can be read from a computer. Coumarin 30 was used for calibrating the set up. The instrument response function (IRF) has been determined from the Raman signal of water to have a width of 110 fs. Lifetimes of fluorescence decay were obtained by fitting the fluorescence decay profile to the most accurate fit. Mono and multi−exponential decay functions convoluted with IRF in MATLAB and Origin 8 were necessary for the data analysis.

Quantum Chemical Simulations. Theoretical investigation has been performed on molecular structures where the long alkyl chains are replaced by short chains (octyl and hexyl groups [C_6H_{13}, C_8H_{17}] attached to the PDI moieties and the BDT linker are replaced by hydrogen atoms and methyl groups, respectively) to save computational time without significant effect on the electronic properties. The ground state geometry of each compound was obtained by density functional theory (DFT). The B3LYP functional and the 6−31G* basis sets have been employed. Excited state simulations using time−dependent DFT (TD-DFT) were performed. The same functional and basis sets used in the ground state calculations, were employed for the geometry optimization of the first singlet excited state (S_1) and the first triplet excited state (T_1) in the gas phase. The geometries of S_1

were obtained with TD-DFT simulations. T_1 geometries could be obtained via two approaches: an unrestricted triplet calculation, and TD-DFT targeting a triplet state. In this study, TD-DFT triplet geometry optimization was conducted for consistency with the S_1 geometries. Single−point energy calculations to evaluate the electronic property were performed using the system−dependent, nonempirically tuned version of long−range corrected functional ωB97X−D[21,22] which is known to significantly improve the charge delocalization problem in conventional DFT functionals and 6−31G* basis sets. The ω value is tuned to minimize the square sum of the difference between HOMO energy (ε_{HOMO}) and ionization potential (IP), and LUMO energy (ε_{LUMO}) and electron affinity (EA), $(\varepsilon_{HOMO}+IP)^2+(\varepsilon_{LUMO}+EA)^2$. The ω value is significantly affected by the environment,[23] and inclusion of the solvent (chloroform) dielectric field induces a reduced ω value. The optimal ω values of **β** and **βC** are 0.004 and 0.003, respectively. The medium effect was included using polarizable continuum model with the dielectric constant of 4.31 for chloroform. Characters of excitations were described with natural transition orbitals (NTOs). The driving force of singlet fission — the energy difference between E(S_1) and 2×E(T_1), $\Delta E_{S\text{-}2\times T}$ — was estimated with the same computational details mentioned above. S_1 and T_1 energies should be calculated at the optimized states since the vibrational relaxation is much faster than SF process. Recently, Krylov et al. pointed out the importance of ^{1}(TT) energy in predicting feasibility of SF and relevant kinetics.[24] Restricted active space with double spin-flip (RAS-2SF) has successfully provided the energetics of SF-relevant states in a number of studies.[25–32] While the RAS-2SF approach works well for dichromophoric systems, **β** and **βC** trimeric compounds require one additional spin-flip to locate all available multiexcitonic states distributed over three chromophores. In this study, we employed a high spin restricted open Hartree-Fock septet reference state. The orbital space of interest was divided into three parts: RAS1, RAS2 and RAS3. Two subspaces, RAS1 and RAS3, correspond to fully occupied, and virtual spaces, respectively. Six singly occupied orbitals were considered in the RAS2 active space. Core inactive occupied orbitals and 1200 virtual orbitals were kept frozen to enable RAS-3SF calculation for such large chromophores. The localized frontier orbitals were obtained using RAS-3SF method via Pipek-Mezey localization scheme with Q-Chem 4.0.[33] Energies of multiexciton state were estimated at the S_1 geometries obtained by the TD-DFT calculation described above. Details on the procedure of intersystem crossing calculations are provided in Supporting Information. All the quantum chemical simulations were conducted using Q−Chem 5.0.

Device fabrication and characterization. Polymer PTB7−Th was obtained from *1−Material*. Chlorobenzene and 1,8−diiodooctane for active layer solution preparation were obtained from Sigma-Aldrich. For ZnO preparation using sol−gel approach, we used $Zn(CH_3COO)_2{\cdot}2H_2O$, 2-methoxyethanol and 2-aminoethanol, all purchased from Sigma−Aldrich. Chloroform and cyclohexane from Sigma−Aldrich were used as solvents for the spectral and photophysical characterization. All chemicals were used as obtained from the manufacturer without further purification. Devices were fabricated in inverted configuration consisting of ITO/ZnO/active layer/MoO_3/Ag. ITO substrates, obtained from Thin Film Devices Inc., were ultrasonicated in chloroform, acetone and isopropanol for 15 min and then treated with UV−ozone for 30 min. Sol−gel solution of ZnO precursor, prepared following procedures described elsewhere, was added dropwise onto ITO substrates through PTFE syringe filter and spin coated at 4000 rpm for 40 seconds. Immediately after spin coating, substrates were annealed at 200^0C for 30 minutes in air. Active layer components were dissolved in chlorobenzene overnight at 70^0C and solution at room temperature was spin coated onto the substrates in a glovebox. Films were immediately transferred to a vacuum chamber and MoO_3 (8 nm) and Ag (80 nm) were thermally evaporated under the pressure lower than $2 \cdot 10^{-6}$ Torr.

J−V curves of the devices were measured with Keithley 2420 source meter unit. Devices were tested under 1 sun conditions (AM1.5G, 100 mW/cm^2) using xenon lamp (Oriel 69920) intensity of which

was calibrated with a standard NREL certified Si cell (Newport, 91150V). Masks with a well−defined area of 3.14 mm^2 were used to define an active area of the device.

2. Steady–State and Two–Photon Absorption Measurements

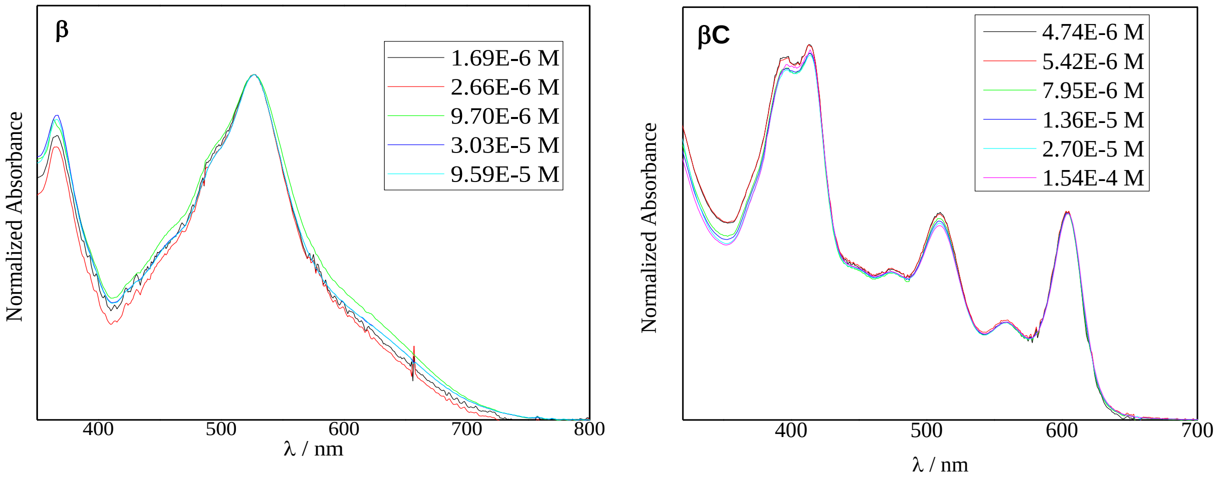

Figure S1. Concentration effect on the absorption spectra of the Trimers in chloroform.

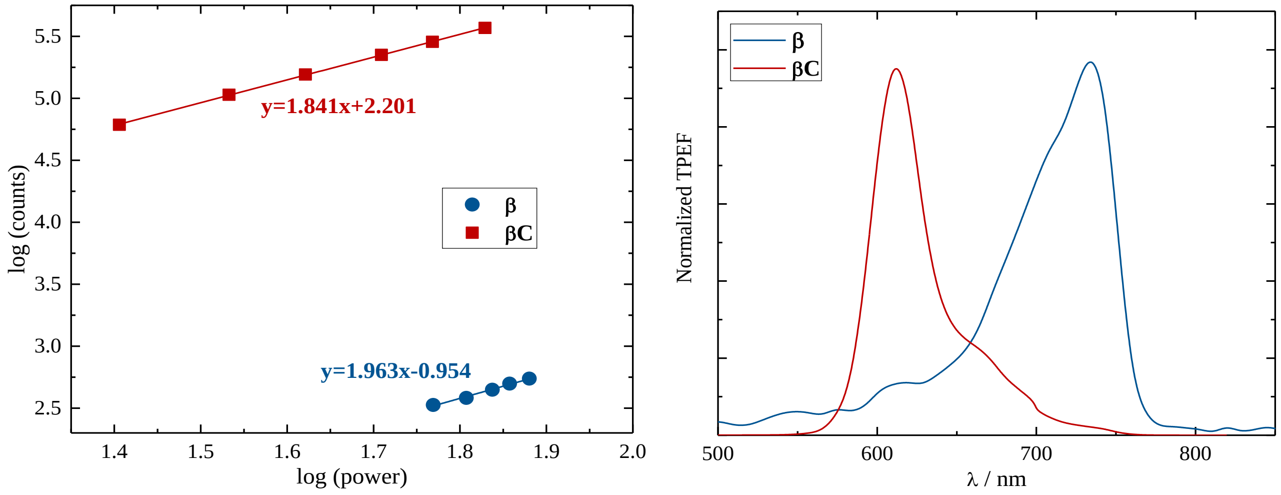

Figure S2. Power dependence of the two−photon excited emission (left) and two−photon emission spectra (right) for the Trimers in chloroform upon 820 nm excitation.

Table S1. Change in atomic charge from S_0 to S_1 on the subunits of compound **β** and **βC**.[a]

	β					βC				
	A	D	A	D	A	A	D	A	D	A
Absorption[b]	−0.07	+0.50	−0.66	+0.27	−0.03	−0.19	+0.32	−0.25	+0.32	−0.19
Emission[c]	+0.07	+0.64	**−0.74**	+0.03	0.00	**−0.79**	+0.61	+0.10	+0.08	**−0.01**

[a]Mulliken charge is given in e^{-}; [b]Calculated at S_0 geometry; [c]Calculated at S_1 geometry

3. Femtosecond Transient Absorption (fsTA)

Table S2. Lifetimes (τ) obtained by global fitting of the femtosecond Transient Absorption data in chloroform.

Compound	τ_1 / ps	τ_2 / ps	τ_3 / ps	τ_4 / ps
**β**	0.18	7.4	320	Rest
**βC**	0.14	33	1300	Rest
assignment	*Solv. / ICT*	*VC/SR*	*S_1*	*T*

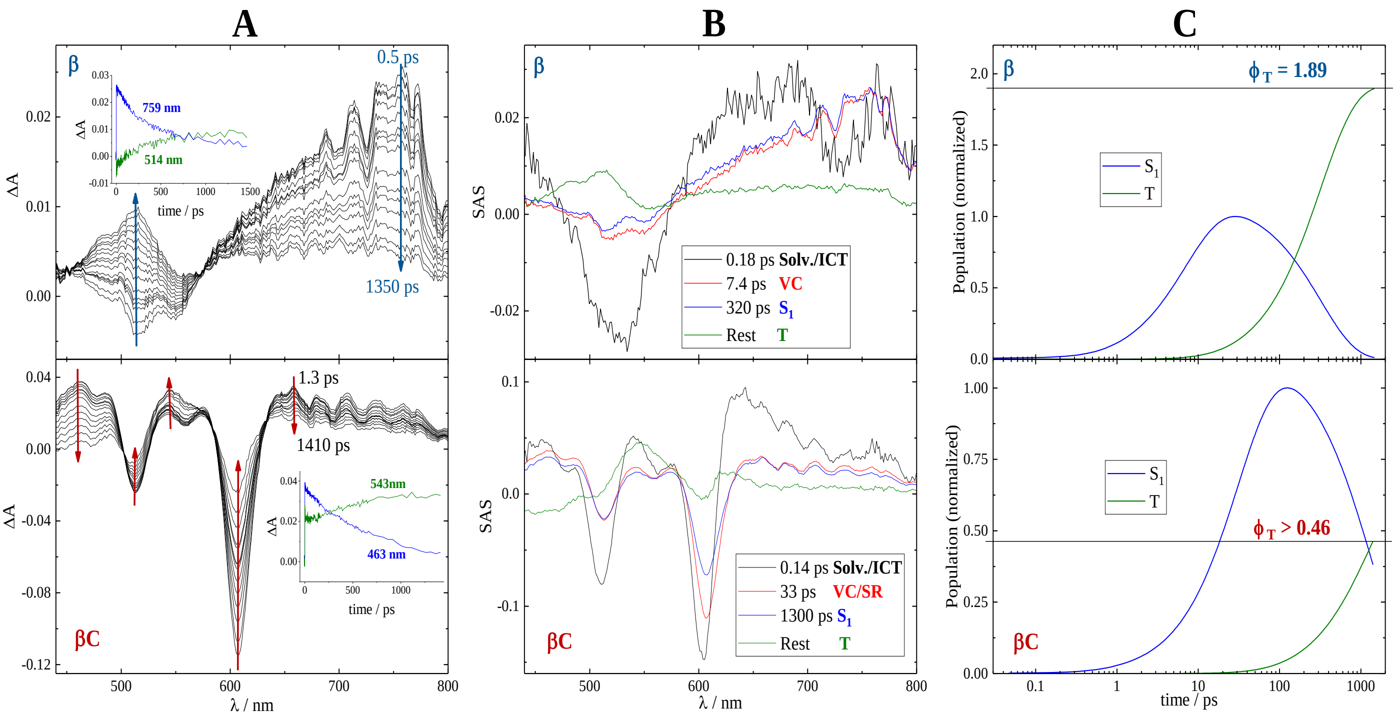

Figure S3. A) Time−resolved spectra and kinetics (inset) obtained by femtosecond TA for the Trimers in chloroform. B) Species Associated Spectra and lifetimes obtained by global fitting the TA data. C) Population dynamics of the excited singlet and triplet states.

4. Triplet Yield Calculation from fsTA

Triplet yield was evaluated from the temporal dynamics of the lowest excited singlet and triplet state populations, as obtained through analysis of the femtosecond transient absorption data, according to a procedure already described in the literature, and here detailed for the trimer samples.[34,35]

βC Trimer

Global Fitting of the femtosecond transient absorption data was carried out using the Glotaran software which provided us with the Species Associated Spectra of the four exponential components (assignments and lifetimes described in Table S2) and their temporal composition (Figure S4).

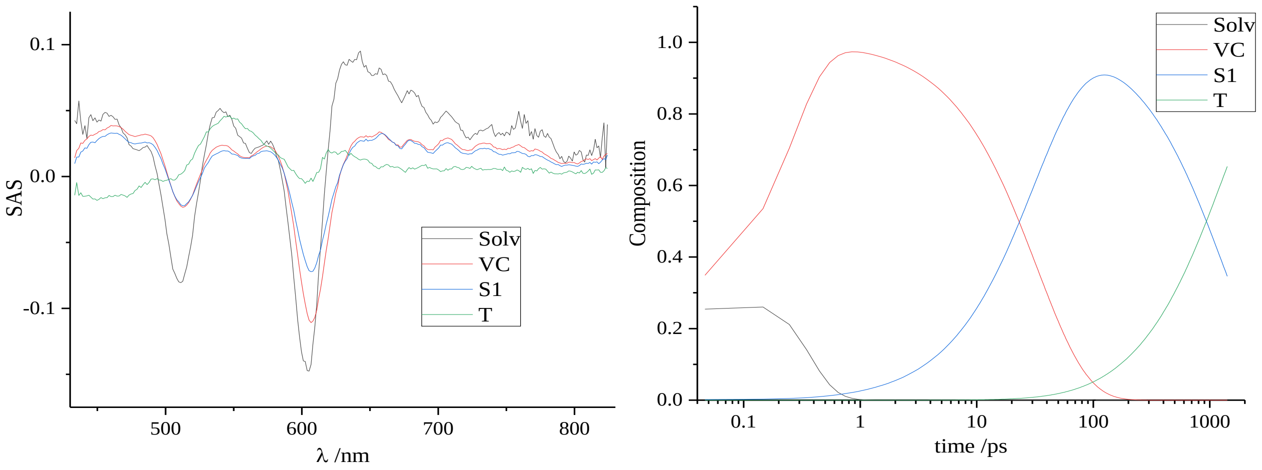

Figure S4. Species Associated Spectra (left) and composition in time (right) of the four exponential components resulting from global fitting of the femtosecond transient absorption data of **βC** in chloroform.

To obtain the spectral shapes of the excited singlet and triplet states the transient spectra at time delays of 129 and 1413 ps were selected, respectively (Figure S5). In fact, at these time delays abundances of the singlet and triplet transients were at the maximum in the femtosecond transient absorption data (Figure S4).

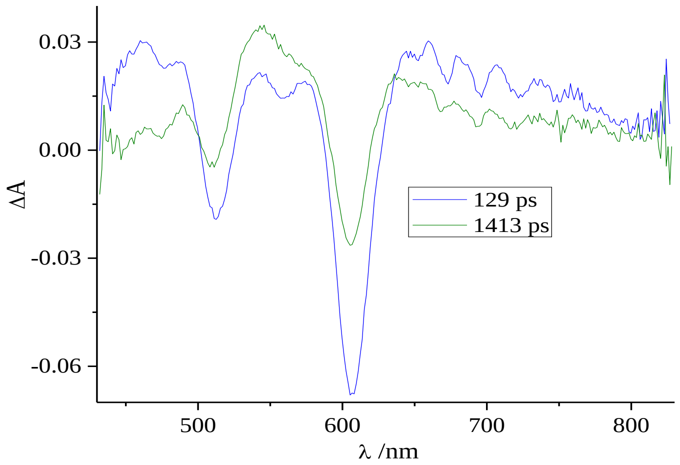

Figure S5. Transient absorption spectra at 129 and 1413 ps delay from excitation recorded for **βC** in chloroform.

The spectra of the lowest singlet and triplet excited states must be related through the GSB they share in common. The ground state absorption spectrum was scaled and subtracted from the 129/1413 ps transient spectra in order to remove the GSB contribution. The ground state absorption spectrum was normalized to the transient absorption spectrum at the peak of the ground state bleaching, and then subtracted. The normalization was the method employed to determine the "right" amount of ground state absorption to subtract each time. The resulting spectra only show the S1/T1 ESA relative to a known amount of GSB (Figure S6).

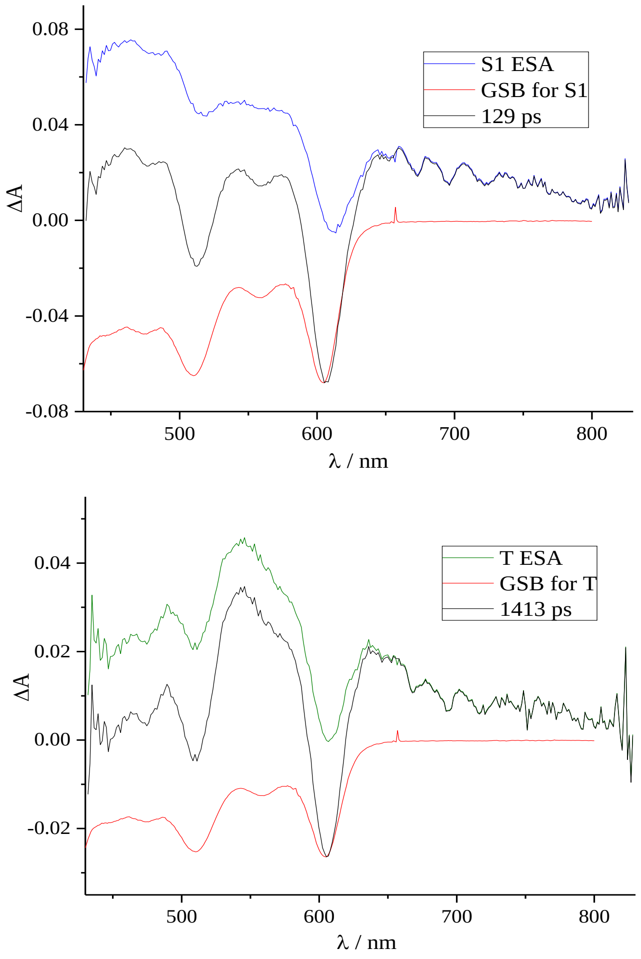

Figure S6. Transient (black) and steady state (red) absorption spectra used to reconstruct the absorption spectra of the excited singlet (blue, left) and triplet (green, right) states.

The S1 and T spectra are then normalized to the GSB they share, resulting in two spectra that are quantitatively related (Figure S7).

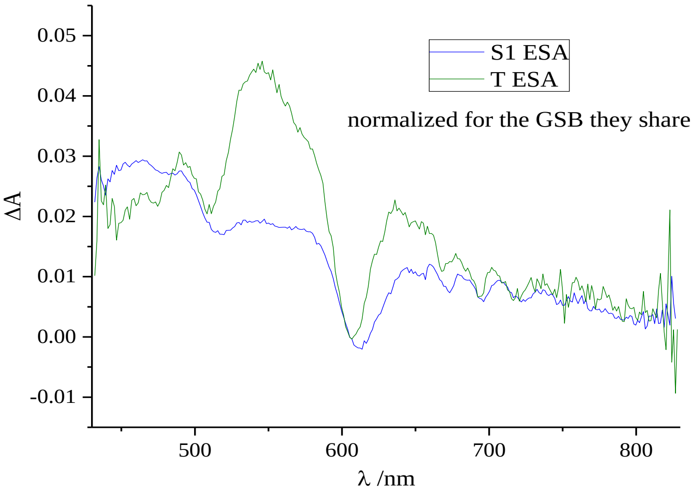

Figure S7. Excited state absorption spectra, normalized to GSB, used to correct composition profile in Figure S4 and to obtain population dynamics in Figure S8.

The quantitatively related spectra of S1 and T were used to correct the composition profiles previously shown. The temporal composition of the components resulting from the global fitting reported in Figure S4 is indeed related to the differential absorbance measured during the ultrafast absorption experiments. Therefore,

according to the Lambert−Beer law, it is dependent on both the absorption ability and the concentration of S1 and T. From the quantitatively related S1 and T spectra reported in Figure S7, it is clear that the ratio between the triplet excited state absorption at its peak (0.0454 at 540 nm) and the singlet excited state absorption at its peak (0.0292 at 460 nm) is $\frac{\varepsilon_T}{\varepsilon_S} = 1.55$. By scaling the S1 and T temporal compositions in Figure S11 for this factor (multiplying the singlet profile by 1.55), the correct concentration profiles were obtained: $\frac{c_S}{c_T} = \frac{\Delta A_S}{\Delta A_T} \times \frac{\varepsilon_T}{\varepsilon_S}$. From the population profiles normalized at the singlet population peak (Figure S8), a triplet quantum yield **higher than 46%** was estimated for **βC**.

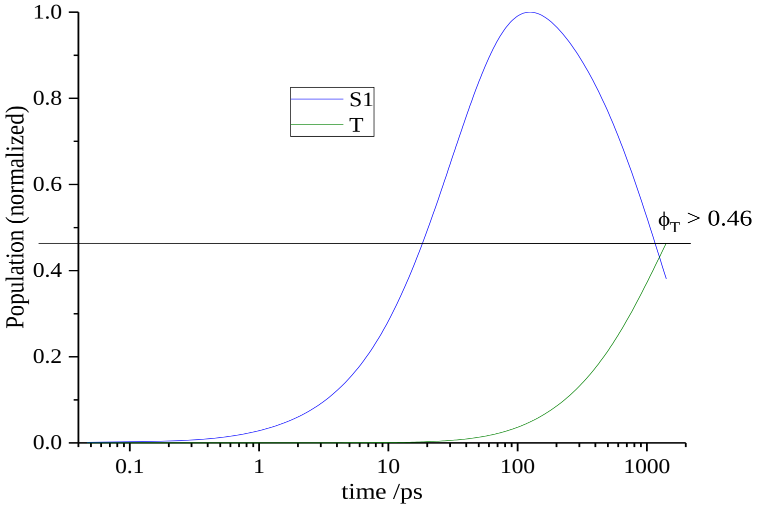

Figure S8. Population dynamics of the excited singlet and triplet states for **βC** in chloroform.

*Note: For **βC**, from the fs transient data we estimate a triplet yield somehow higher than that accurately measured by the ns sensitization experiments. However, all the methods agree in giving a triplet yield lower than 100 % for the **βC** compound, clearly suggesting a triplet production via conventional ISC.*

<u>β Trimer</u>

A similar procedure was employed to evaluate the triplet yield for **β**. Global Fitting revealed the presence of four exponential components whose Species Associated Spectra and composition in time are shown below in Figure S9.

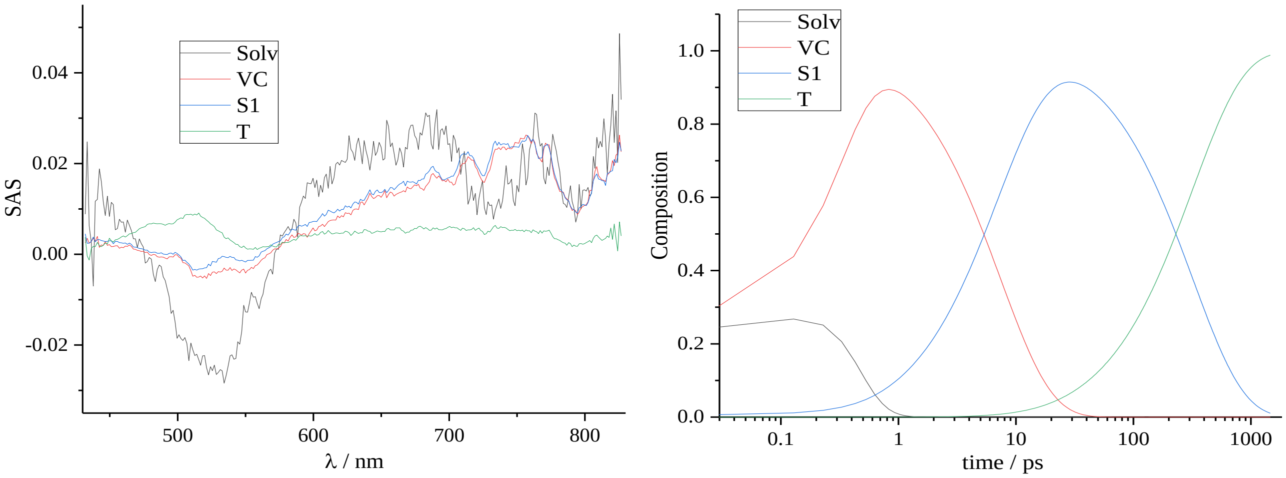

Figure S9. Species Associated Spectra (left) and composition in time (right) of the four exponential components resulting from global fitting of the femtosecond transient absorption data of **β** in chloroform.

Looking at the singlet and triplet composition profiles, transient spectra at time delays of 29 and 1165 ps were selected to obtain spectral shapes associated to the excited singlet and triplet state, respectively (Figure S10).

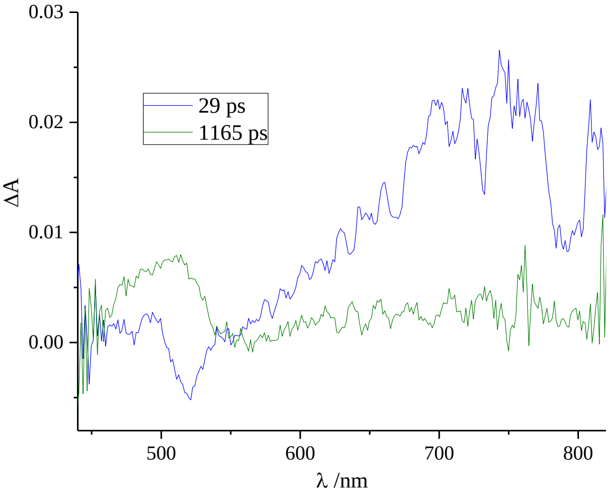

Figure S10. Transient absorption spectra at 29 and 1165 ps delay from excitation recorded for **β** in chloroform.

The ground state absorption spectrum was scaled and subtracted from the 29/1165 ps transient spectra in order to remove the GSB contribution (Figure S11). In this case the same amount of GSB was subtracted from both spectra because no clear negative band is exhibited by the transient spectrum at long delays. This is due to spectral overlap between the ground state and the triplet excited state absorption.

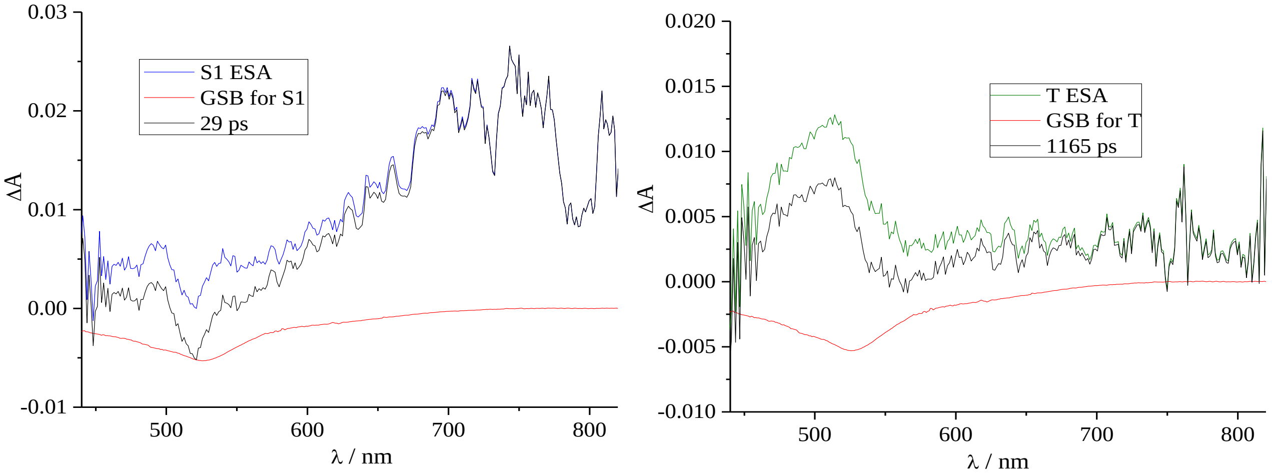

Figure S11. Transient (black) and steady state (red) absorption spectra used to reconstruct the absorption spectra of the excited singlet (blue, left) and triplet (green, right) states.

The resulting spectra, which only show S1/T ESA signals peaked at very different wavelengths, are quantitatively related (Figure S12).

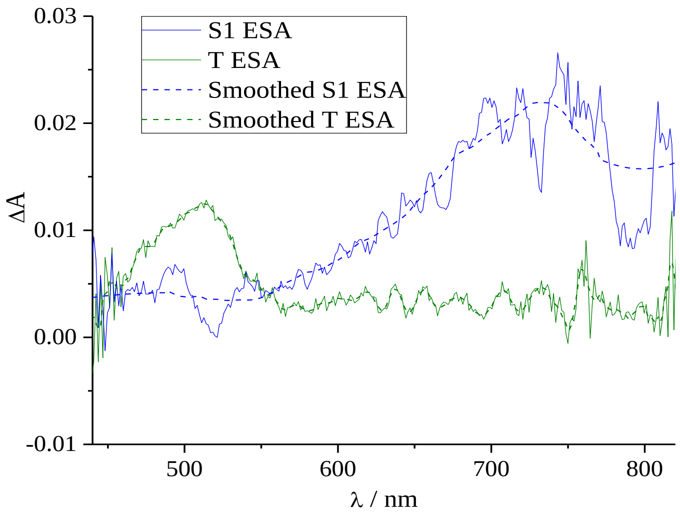

Figure S12. Excited state absorption spectra used to correct composition profile in Figure S9 and to obtain population dynamics in Figure S13.

These spectra were used to correct the composition profiles shown in Figure S9, after a smoothing procedure applied to be more accurate in determining differential absorbance typical of singlet and triplet states. The ratio between the triplet excited state absorption at its peak (0.0125 at 510 nm) and the singlet excited state absorption at its peak (0.0219 at 740 nm) is 0.571. The temporal population profiles for these excited states were thus obtained, by scaling the composition profiles in Figure S9 using this factor (multiplying the singlet profile by 0.571). Population dynamics normalized at the singlet peak (Figure S13) demonstrates the formation of 1.89 triplets per initially excited singlet. A triplet quantum yield of **189%** was estimated for **β**.

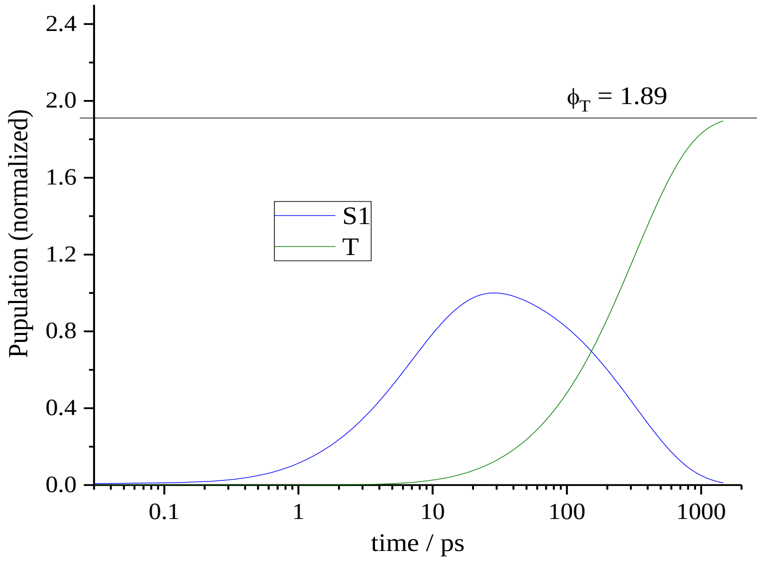

Figure S13. Population dynamics of the excited singlet and triplet states for **β** in chloroform.

5. Nanosecond Transient Absorption (nsTA)

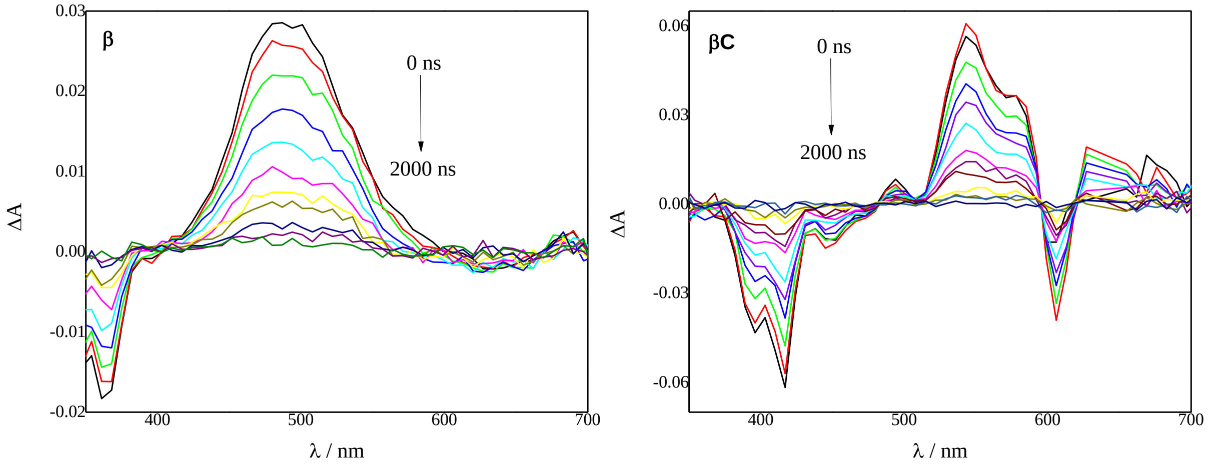

Figure S14. Time−resolved spectra obtained by nanosecond TA measurements for the Trimers in air-equilibrated chloroform upon 500 nm (for **β**) and 600 nm (for **βC**) laser excitations.

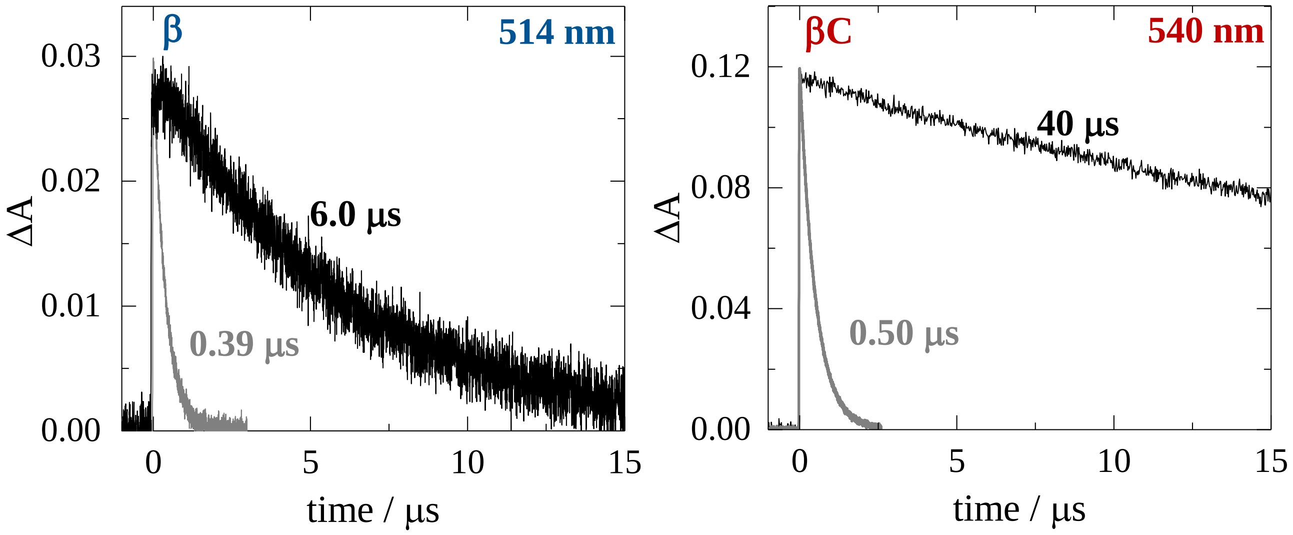

Figure S15. Kinetics of triplet species in air-equilibrated (gray) vs. deaerated (black) chloroform.

6. Triplet Sensitization Experiments by nsTA

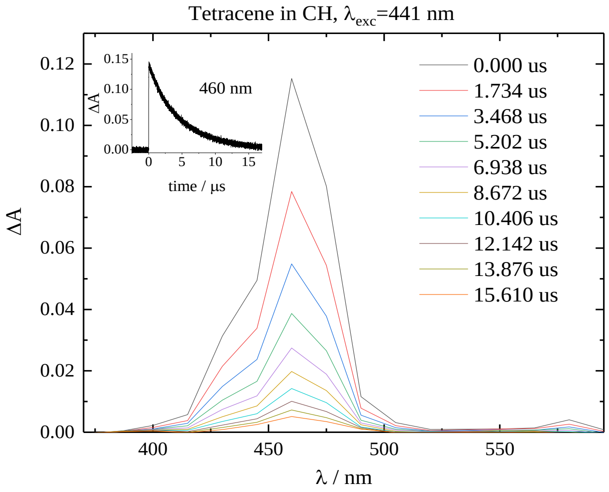

Figure S16. Transient absorption spectra of Tetracene in cyclohexane upon laser excitation at 441 nm.

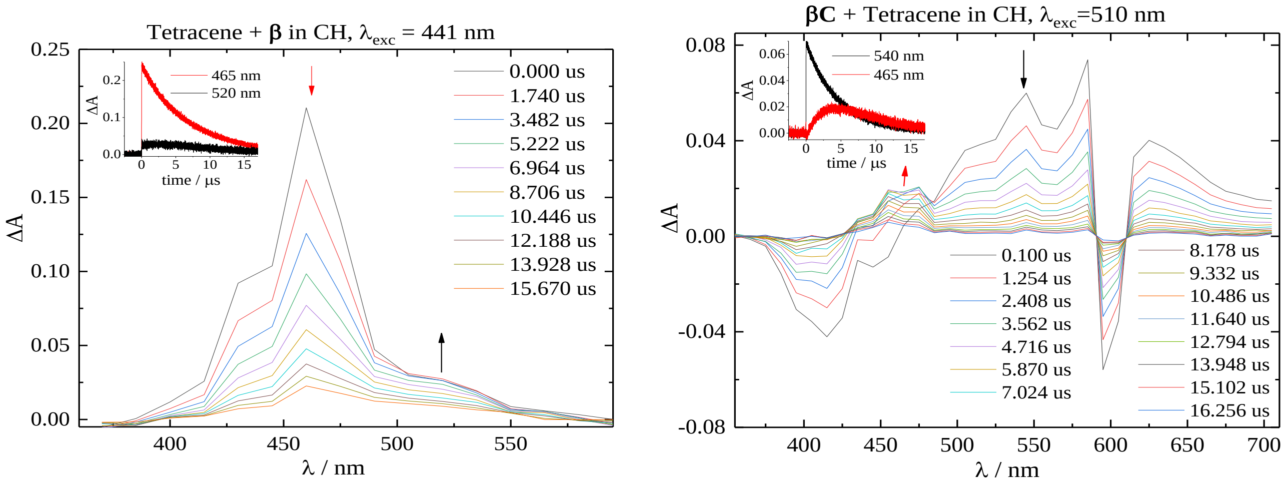

Figure S17. Transient absorption sensitization spectra of the Trimer compounds and Tetracene in cyclohexane, showing the triplet species decay of the donor and the rise of the acceptor (inset).

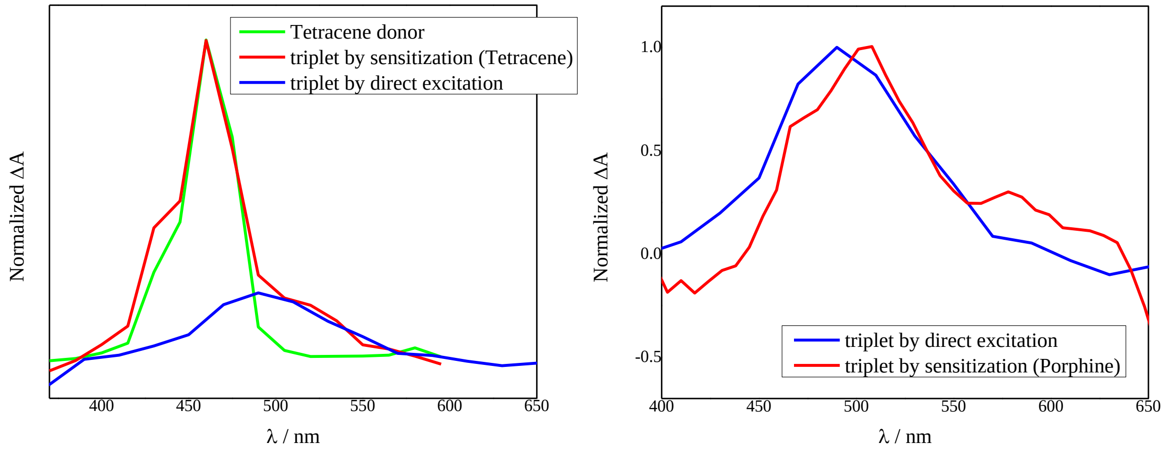

Figure S18. Triplet absorption spectrum of β from direct excitation and from sensitization by using Tetracene (left) and tetraphenyl-Porphine (right) as triplet donor.

Triplet extinction coefficient of **β** was obtained by triplet energy transfer from Tetracene donor (E_T = 1.27 eV) to the **β** triplet energy acceptor (E_T << 1.27 eV). For **βC**, its triplet extinction coefficient was obtained by energy transfer to the Tetracene acceptor from **βC** acting as a triplet energy donor (E_T > 1.27 eV).
Here, the employed procedures for the two trimer compounds are reported.

1) Determination of the triplet extinction coefficient of β in cyclohexane (CH) at 520 nm by energy transfer from Tetracene (λ_{exc} = 441 nm)[12,14–16]

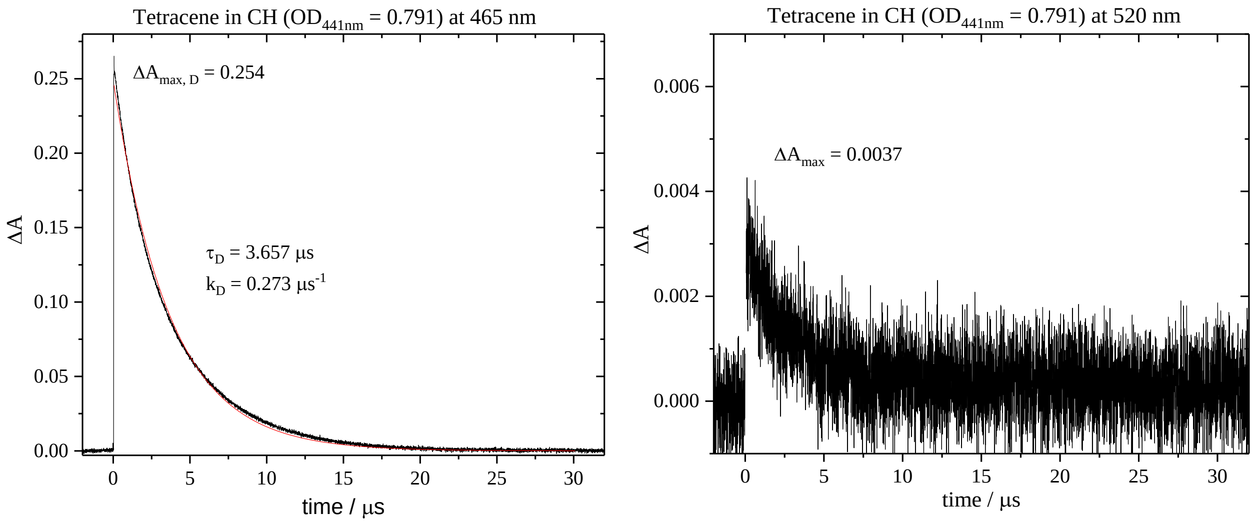

Figure S19. Kinetics recorded during nanosecond transient absorption measurements of **Tetracene** (donor) in cyclohexane upon laser excitation at 441 nm.

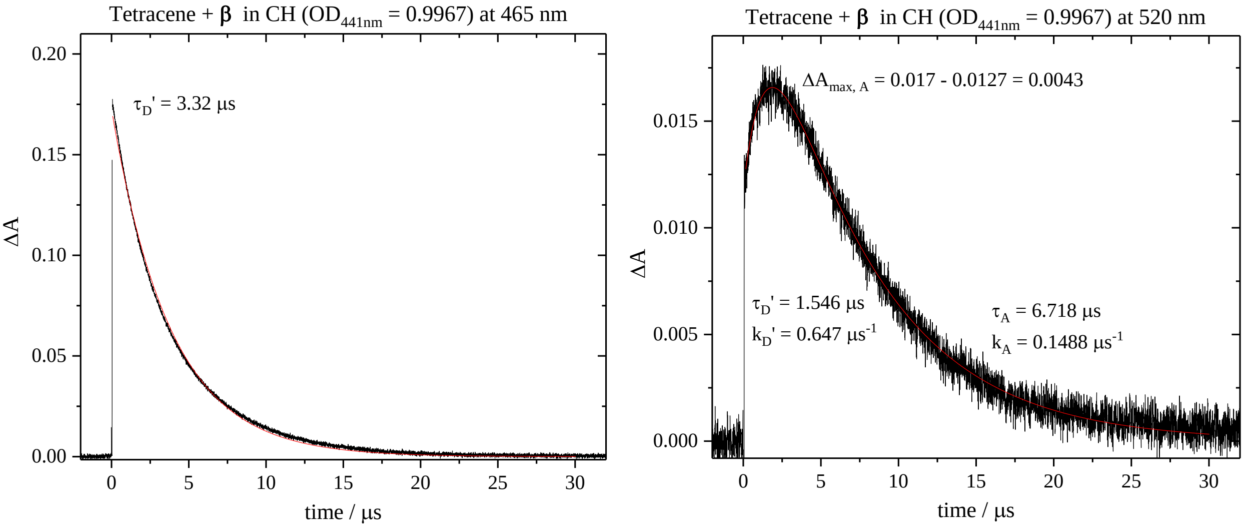

Figure S20. Kinetics recorded during nanosecond transient absorption measurements of **β** sensitized by **Tetracene** (quenched donor at 465 nm and sensitized acceptor at 520 nm) in cyclohexane upon laser excitation at 441 nm.

$$\varepsilon_A = \varepsilon_D \times \frac{\Delta A_{max,A}}{\Delta A_{max,D}} \times \frac{1}{f_D \times p_{ET} \times w}$$

$$\varepsilon_A = 31200\ M^{-1}cm^{-1} \times \frac{0.0043}{0.254} \times \frac{1}{0.8514 \times 0.5773 \times 0.6446} = 1667\ M^{-1}cm^{-1}$$

Where:

$f_D = \frac{A_D}{A_{D+A}} \times \left(\frac{1-10^{-A_{D+A}}}{1-10^{-A_D}}\right) = 0.8514$ is the fraction of light absorbed by the donor

$p_{ET} = \frac{k'_D - k_D}{k'_D} = 0.5773$ is the energy transfer probability

$w = exp\left[-\frac{\ln\left(\frac{k'_D}{k_A}\right)}{\frac{k'_D}{k_A}-1}\right] = 0.6446$ is a factor accounting for the quenched donor and acceptor lifetimes

And where,

ε_A = triplet-triplet extinction coefficient of the acceptor (UNKNOWN)

ε_D = triplet-triplet extinction coefficient of the donor/sensitizer (KNOWN)

$\Delta A_{max,A}$ = maximum absorbance change of the Acceptor in the "Donor + Acceptor" mixture

$\Delta A_{max,D}$ = maximum absorbance change of the Donor/sensitizer **alone**

f_D = fraction of light absorbed by the Donor/sensitizer in the "Donor + Acceptor" mixture w.r.t. the Donor alone

k'_D = rise rate constant of the Acceptor in the "Sensitizer + Acceptor" mixture (should be similar to the quenched Sensitizer decay rate constant)

k_D = decay rate constant of the Donor ONLY

k_A = decay rate constant of the Acceptor in the "Donor + Acceptor" mixture

*Note: In estimating k'_D, ideally (in the absence of any spectral overlap) the decay rate constant of the quenched Donor (at 465 nm) should match the rise rate constant of the Acceptor (at 520 nm).[15] This was not the case here for the **β** compound as there is an overlap of its triplet spectrum and that of the tetracene Donor such that at 465 nm both the Donor and the Acceptor contribute to the decay rate constant (see Figures 4 and S15). For this reason, we decided to set k'_D as the rise rate constant of the Acceptor because at 520 nm only the **β** compound triplet shows an absorption.*

To calculate the Triplet quantum yield.

Relative Actinometry approach was used.[11] This was done using the computed triplet extinction coefficient from the energy transfer measurement. Tetracene, with its known ε_T and ϕ_T, was used as a reference compound (ref.). In order to obtain the ΔA of both sample and reference, the ground state absorption (i.e. OD) of both sample and reference has to be the same at the excitation wavelength (441 nm).

$$\frac{[\phi_T \varepsilon_T]_{sample\ (\beta)}}{[\phi_T \varepsilon_T]_{ref.}} = \frac{[\Delta A]_{sample\ (\beta)}}{[\Delta A]_{ref.}}$$

$$[\phi_T \varepsilon_T]_{sample\ (\beta)} = [\phi_T \varepsilon_T]_{ref.} \times \frac{[\Delta A]_{sample\ (\beta)}}{[\Delta A]_{ref.}} = (0.62 \cdot 31200 M^{-1}cm^{-1}) \times \frac{0.0527}{0.366} = 2785\ M^{-1}cm^{-1}$$

Where,

$\varepsilon_{T\ sample\ (\beta)}$ = triplet-triplet extinction coefficient of the **β** sample (computed using ***energy transfer meas.***)

$\phi_{T\ sample\ (\beta)}$ = triplet quantum yield of the sample **β**

$[\Delta A]_{sample\ (\beta)}$ = change in absorption of the sample **β** (whose OD is similar to that of the reference at $\lambda_{excitation}$)

$[\Delta A]_{ref}$ = change in absorption of the reference compd. (whose OD is similar to that of the sample at $\lambda_{excitation}$)

Table S3. Summary of the parameters recorded for the two triplet energy transfer measurements performed for **β** in CH to ensure reproducibility. Tetracene used as a triplet energy donor.

Parameters	Measurement 1	Measurement 2
$\varepsilon_D(Donor)$	31200 $M^{-1}cm^{-1}$ (Tetracene)	31200 $M^{-1}cm^{-1}$ (Tetracene)
ΔA_A	0.0043	0.005
ΔA_D	0.254	0.170
f_D	0.8514	0.746
k_D	0.273 μs^{-1}	0.175 μs^{-1}
$k'_D = k_D + k_{ET}[A]$	0.647 μs^{-1}	1.919 μs^{-1}
k_A	0.149 μs^{-1}	0.109 μs^{-1}
$\varepsilon_A = \varepsilon_{T\ sample}\ (\beta)$	1667 $M^{-1}cm^{-1}$	1607 $M^{-1}cm^{-1}$
$[\varepsilon_T \cdot \phi_T]_{sample}\ (\beta)$ (Rel. Actinom. meas.)	2785 $M^{-1}cm^{-1}$	2785 $M^{-1}cm^{-1}$
$\phi_{T\ sample}\ (\beta)$	**1.67**	**1.73**

Taking an **average** of these two measurements, the triplet yield was evaluated to be: ~ **1.70**

2) Determination of the triplet extinction coefficient of βC in cyclohexane (CH) at 540 nm by energy transfer to Tetracene (λ_{exc}=510 nm)

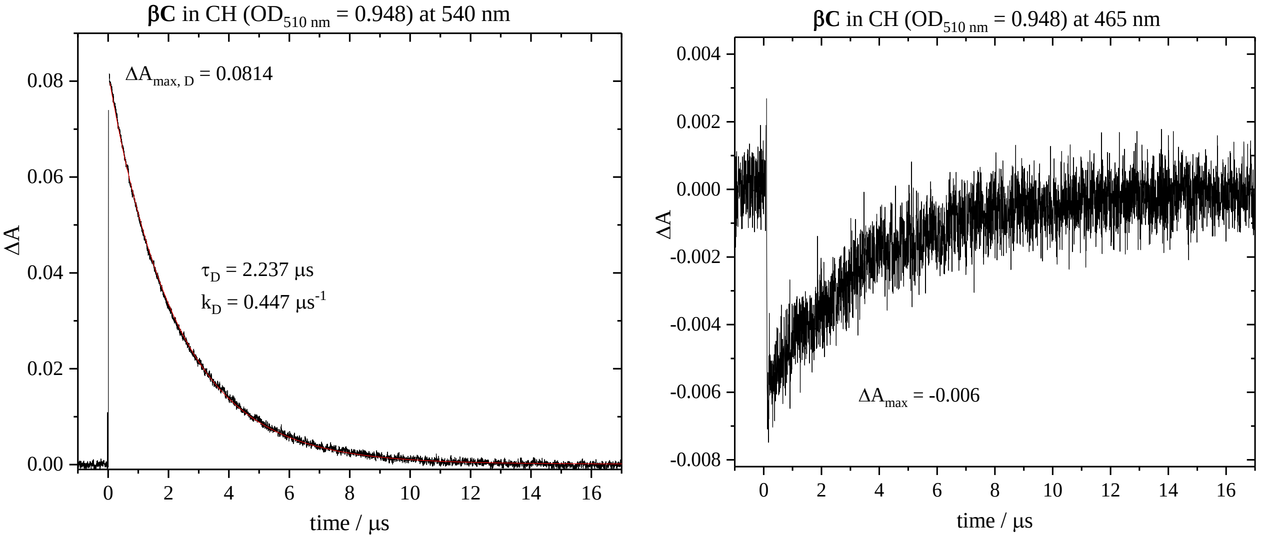

Figure S21. Kinetics recorded during nanosecond transient absorption measurements of **βC** (donor) in cyclohexane upon laser excitation at 510 nm. *It should be noted that the difference in the lifetime of **βC** in comparison to that reported in Table 2 of the main paper is due to the different solvent employed here and to the different nitrogen purging conditions (time, flow rate, ...).*

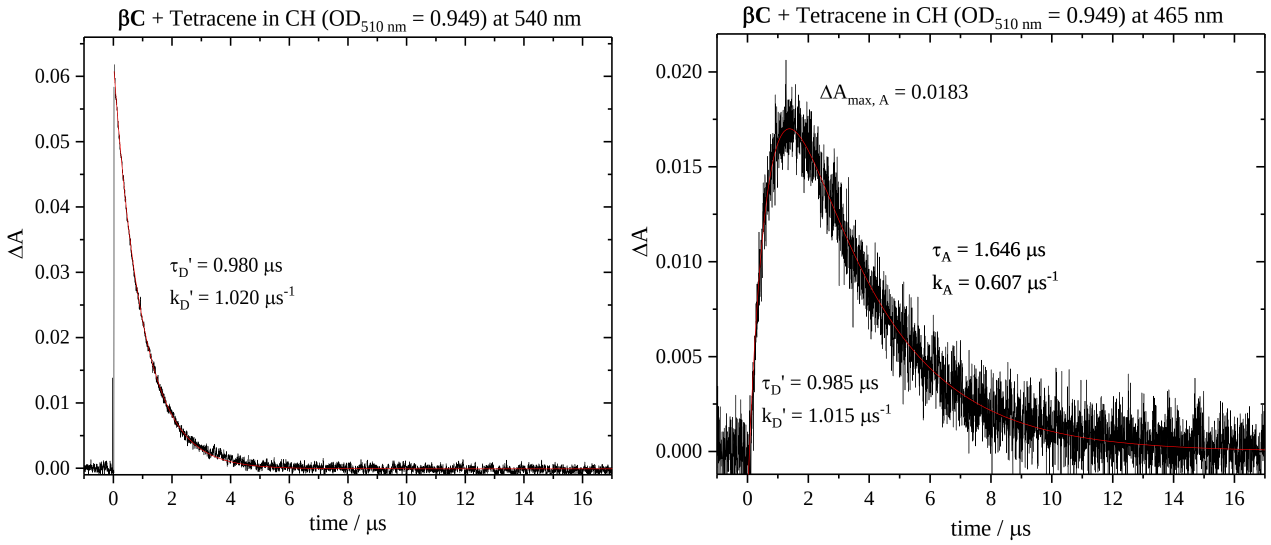

Figure S22. Kinetics recorded during nanosecond transient absorption measurements of Tetracene sensitized by **βC** (quenched donor at 540 nm and sensitized acceptor at 465 nm) in cyclohexane upon laser excitation at 510 nm. *The difference in the lifetime of* Tetracene *in comparison to that reported in Figure S15 is due to the non-consistent nitrogen purging conditions (time, flow rate, ...).*

$$\varepsilon_D = \varepsilon_A \times \frac{\Delta A_{max,D}}{\Delta A_{max,A}} \times f_D \times p_{ET} \times w = 31200\ M^{-1}cm^{-1} \times \frac{0.0814}{0.0183} \times 0.9985 \times 0.5594 \times 0.4652$$
$$= 36061\ M^{-1}cm^{-1}$$

Where:

$f_D = \frac{A_D}{A_{D+A}} \times \left(\frac{1-10^{-A_{D+A}}}{1-10^{-A_D}}\right) = 0.9985$ is the fraction of light absorbed by the donor

$p_{ET} = \frac{k'_D - k_D}{k'_D} = 0.5594$ is the energy transfer probability

$w = exp\left[-\frac{\ln\left(\frac{k'_D}{k_A}\right)}{\frac{k'_D}{k_A}-1}\right] = 0.4652$ is a factor accounting for the quenched donor and acceptor lifetimes

*<u>Note:</u> Here, in estimating k'_D, the decay rate constant of the quenched Donor (at 540 nm) matches the rise rate constant of the Acceptor (at 465 nm). This is because there is no overlap of the triplet absorption spectrum of the **βC** compound and that of the tetracene Acceptor (see Figures 3 and S6), unlike in the case of the **β** compound.*

$$\frac{[\phi_T \varepsilon_T]_{sample\ (\beta C)}}{[\phi_T \varepsilon_T]_{ref.}} = \frac{[\Delta A]_{sample(\beta C)}}{[\Delta A]_{ref.}}$$

$$[\phi_T \varepsilon_T]_{sample\ (\beta C)} = [\phi_T \varepsilon_T]_{ref.} \times \frac{[\Delta A]_{sample\ (\beta C)}}{[\Delta A]_{ref.}} = (0.62 \cdot 31200 M^{-1}cm^{-1}) \times \frac{0.125}{0.324} = 7460 M^{-1}cm^{-1}$$

Table S4. Summary of the parameters recorded for the two triplet energy transfer measurements performed for **βC** in CH to ensure reproducibility. Tetracene used as a triplet energy acceptor in this case.

**Parameters**	**Measurement 1**	**Measurement 2**
ε_A $(\boldsymbol{Acceptor})$	$31200\ M^{-1}cm^{-1}$ (Tetracene)	$31200\ M^{-1}cm^{-1}$ (Tetracene)
ΔA_D	0.0814	0.086
ΔA_A	0.0183	0.012
f_D	0.9985	1
k_D	0.4470 μs^{-1}	0.2476 μs^{-1}
$k'_D = k_D + k_{ET}[A]$	1.0145 μs^{-1}	0.7158 μs^{-1}
k_A	0.6074 μs^{-1}	0.4063 μs^{-1}
$\varepsilon_D = \varepsilon_{T\ sample\ (\beta C)}$	$36061\ M^{-1}cm^{-1}$	$69545\ M^{-1}cm^{-1}$
$[\varepsilon_T \cdot \phi_T]_{sample\ (\beta C)}$ (Rel. Actinom. meas.)	$7460\ M^{-1}cm^{-1}$	$7460\ M^{-1}cm^{-1}$
$\phi_{T\ sample\ (\beta C)}$	**0.21**	**0.11**

Taking an **average** of these two measurements, the triplet yield was evaluated to be: ~ **0.16**

7. Two–Color Transmission Measurement of Triplet Species to compute Triplet Yield

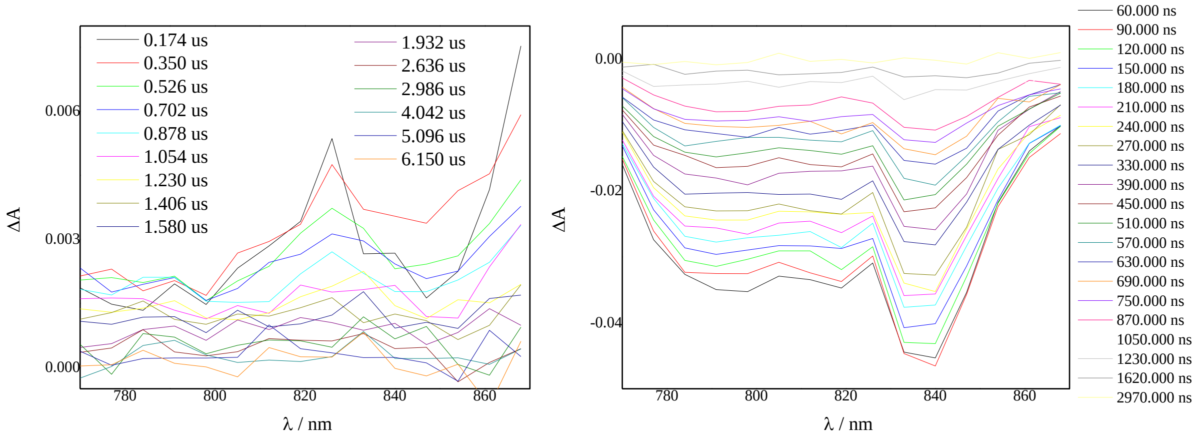

Figure S23. Time resolved absorption spectra recorded in the region between 770 and 870 nm via nanosecond transient absorption for **β** (left) and **βC** (right) in chloroform upon 510 nm laser excitation.

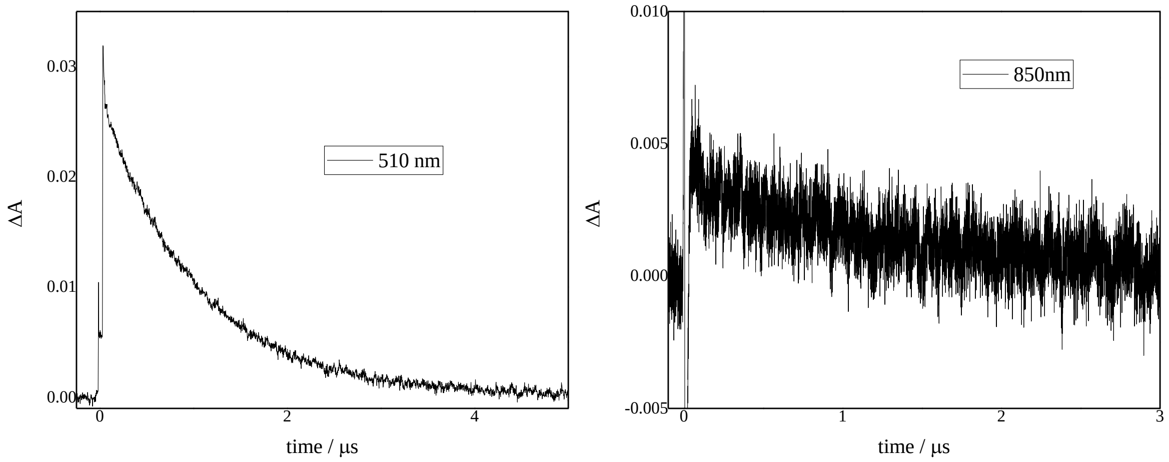

Figure S24. Kinetics recorded at 510 nm (left) and 850 nm (right) for **β** in chloroform via nanosecond transient absorption upon 510 nm laser excitation.

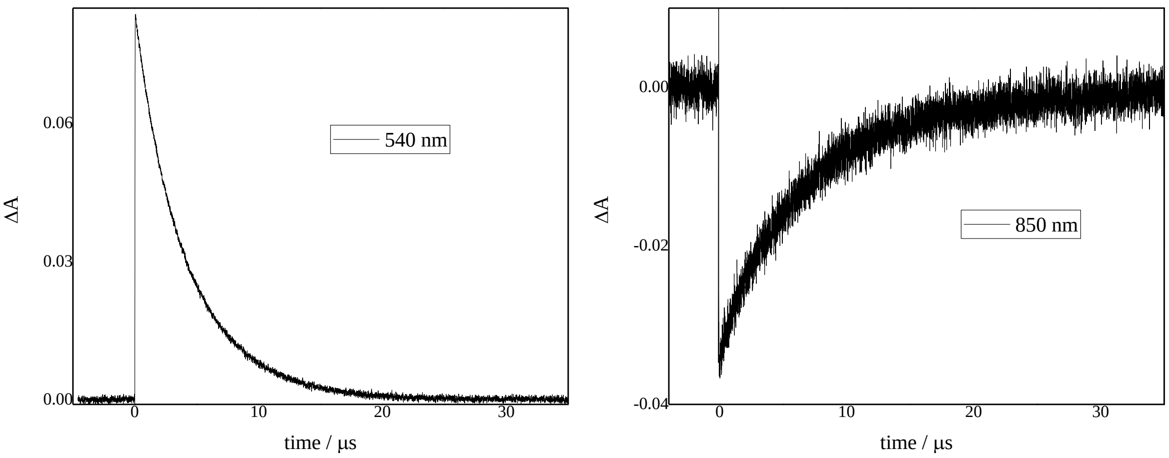

Figure S25. Kinetics recorded at 540 nm (left) and 850 nm (right) for **βC** in chloroform via nanosecond transient absorption upon 510 nm laser excitation.

Using the laser parameters and the 850nm probe beam attenuation caused by the 425nm pump, one can estimate the singlet to triplet conversion efficiency in the trimers. Following information outlines the calculation of singlet-triplet conversion efficiency from experimental data. The triplet extinction coefficient for the trimers at 850 nm was evaluated via nanosecond transient absorption measurements (see Figures S22, S23 and S24). Triplet extinction coefficients at 850 nm of 280 M^{-1} cm^{-1} for **β** and of 9100 M^{-1} cm^{-1} for **βC** were used in the quantitative analysis of the two-color transmission measurements.

Twisted Trimer β in chloroform

Number of singlet excitations have been created by ultrashort 425nm laser pulse

- Average laser power at 425 *nm* = 4.25 *mW* (probe power at 850 nm ≤1.20 mW, beam attenuation 29.7%)
- Energy per pump pulse = 0.00425/(8×10^7) = 0.053 *nJ/pulse*
- Pulse duration = 130 *fs* = 1.3×10^{-13} *s*

- Laser beam divergence (full angle) : ≤ 1 *mrad* (Mai-Tai specs) = 10^{-3} *rad*
- Focus of the focusing lens = 0.3 *cm*

Waist diameter of focal beam = 0.3 × 10^{-3} *cm* (30 *μm*)
Focal waist area =$\pi D^2/4 = 0.707\times10^{-7}$ *cm²*
Excitation volume = 0.707×10^{-7} cm^2 ×1cm=0.707×10^{-7} cm^3

- Energy density for 425nm pump pulse = $0.053\times10^{-9}/(0.707\times10^{-5}) \approx 7.50\times10^{-6}$ J/cm^2

Energy of a photon at 425 nm = 4.7 × 10^{-19} *J/photon*

<u>Singlet excitations number density:</u>
The number of absorbed photons at 425nm: Optical density of the sample at 425nm = **0.116**
$I_{in}-I_{out} = I_{in}(1-10^{-0.116}) = I_{in}(1-0.76) = 0.23*I_{in}$
$I_{in}=0.053\times10^{-9}/(4.7\times10^{-19})=1.1\times10^{8}$phot/pulse
That is $0.23\times1.1\times10^8 = 0.259\times10^8$ photons have been absorbed per pulse.
The same number of molecules in the excited singlet state has been created in the excitation volume ($v = 0.707\times10^{-5}cm^3$)
Excited singlet number density: $n_S^* = 0.259\times10^8/(0.707\times10^{-5}) = 3.66\times10^{12}$ molecules/cm³

<u>Triplet excitation number density:</u>
850nm - probe beam attenuation observed under pump of 4.25 mW is **29.7 %**
No focal mismatch is suggested in this version of calculations.
In order to create this attenuation for the matching part of the probe beam the concentration of the triplet states responsible for attenuation can be obtained:
$I_{out}/I_{in} = 1 - 0.297 = 0.703 = 10^{-\varepsilon l M_T}$, (ε-extinction coefficient for triplet-triplet absorption, l – cell length, M_T - triplet states concentration) or
$-\varepsilon l M_T=\log(0.703) = -0.153$
$M_T=0.153/(1\times280)=0.546\times10^{-3}$ M where 280 $M^{-1}cm^{-1}$ is the molar extinction coefficient for triplet-triplet absorption
This molar concentration corresponds to the triplet population density
$n_{TE} = N_A\times M_T/1000= 3.29\times10^{17}$ triplets/cm³
Full number of triplet in the excitation volume v is
$N_{TT} = n_{TE}\times v = 3.29\times10^{17} \times0.707\times10^{-7} = 2.32\times10^{10}$ molecules in the triplet state in the excitation volume
Each pulse creates $N_S^* = 0.259\times10^8$ molecules in the singlet state
The number of triplet states created by each pulse is:
$N_{TP}= \alpha\times0.259\times10^8$ molecules in the triplet state in the excitation volume.
Now the triplet state accumulation at relatively high pulse repetition rate should be taken into account:
$N_{Taccum} = N_{TP}\times\tau_T\times8\times10^7$; where τ_T is triplet state lifetime **(~6μs)**, 8×10^7 - laser pulse repetition rate
$N_{Taccum} = 0.259\times10^8\times\alpha\times6\times10^{-6}\times8\times10^7=1.24\times10^{10}\times\alpha$:
$\alpha = (2.32\times10^{10} /1.24\times10^{10})$ = **1.87 or 187%**

Planar Trimer βC in chloroform

<u>Number of singlet excitations have been created by ultrashort 425nm laser pulse</u>

- Average laser power at 425 *nm* = 4.25 *mW* (probe power at 850 nm ≤1.20 mW, beam attenuation 36.6%)
- Energy per pump pulse=$0.00425/(8\times10^7)$ = 0.053 *nJ/pulse*
- Pulse duration = 130 *fs* = 1.3 ×10^{-13} *s*
- Laser beam divergence (full angle) : ≤ 1 *mrad* (Mai-Tai specs) = 10^{-3} *rad*
- Focus of the focusing lens = 0.3 *cm*

Waist diameter of focal beam = 3 × 10^{-3} *cm* (30 *μm*)
Focal waist area = $\pi D^2/4 = 0.707\times10^{-7}$ *cm²*

Excitation volume =0.707×10^{-7} cm^2 ×1cm = 0.707×10^{-7} cm^3

- Energy density for 425nm pump pulse = $0.053\times10^{-9}/(0.707\times10^{-5}) \approx 7.50\times10^{-6}$ *J/cm²*

Energy of a photon at 425 nm = 4.7×10^{-19} *J/photon*

<u>Singlet excitations number density:</u>

The number of absorbed photons at 425nm: Optical density of the sample at 425nm = **0.885**

$I_{in}-I_{out} = I_{in}(1-10^{-0.885}) = I_{in}(1-0.13)=0.87\times I_{in}$

$I_{in}=0.053\times10^{-9}/(4.7\times10^{-19})=1.1\times10^{8}$phot/pulse

That is 0.980×10^{8} photons have been absorbed per pulse.

The same number of molecules in the excited singlet state has been created in the excitation volume (v = 0.707×10^{-5}cm³)

Excited singlet number density: $n_S^* = 0.980\times10^{8}/(0.707\times10^{-5}) = 13.9\times10^{12}$ molecules/cm³

<u>Triplet excitation number density:</u>

850nm - probe beam attenuation observed under pump of 4.25 mW is **36.6 %**

No focal mismatch is suggested in this version of calculations.

In order to create this attenuation for the matching part of the probe beam the concentration of the triplet states responsible for attenuation can be obtained:

$I_{out}/I_{in} = 0.654 = 10^{-\varepsilon lMT}$, (ε-extinction coefficient for triplet-triplet absorption, l – cell length, M_T - triplet states concentration) or

$-\varepsilon lM_T = \log(0.654)=-0.184$

$M_T=0.184/(1\times9100)=0.202\times10^{-4}$ M where 9100 $M^{-1}cm^{-1}$ is the molar extinction coefficient for triplet-triplet absorption

This molar concentration corresponds to the triplet population density

$n_{TE} = N_A\times M_T/1000= 1.22\times10^{16}$ triplets/cm³

Full number of triplet in the excitation volume v is

$N_{TT} = n_{TE}\times v = 1.22\times10^{16} \times0.707\times10^{-7} = 8.61\times10^{8}$ molecules in the triplet state in the excitation volume

Each pulse creates $N_S^* = 0.980\times10^{8}$ molecules in the singlet state

The number of triplet states created by each pulse is:

$N_{TP}= \alpha\times0.980\times10^{8}$ molecules in the triplet state in the excitation volume.

Now the triplet state accumulation at relatively high pulse repetition rate should be taken into account:

$N_{Taccum} = N_{TP}\times\tau_T\times8\times10^{7}$; where τ_T is triplet state lifetime (~**40µs**), 8×10^{7} - laser pulse repetition rate

$N_{Taccum} = 0.980\times10^{8}\times\alpha\times40\times10^{-6}\times8\times10^{7}=3.14\times10^{11}\times\alpha$:

$\alpha = (8.61\times10^{8} /3.14\times10^{11})$ = **0.0027 or 0.27%**

It has to be noted that for the case of the **βC** compound, the estimated triplet yield value is not accurate because of the observed phosphorescence interference at 850 nm.

8. Fluorescence Up-Conversion (FUC)

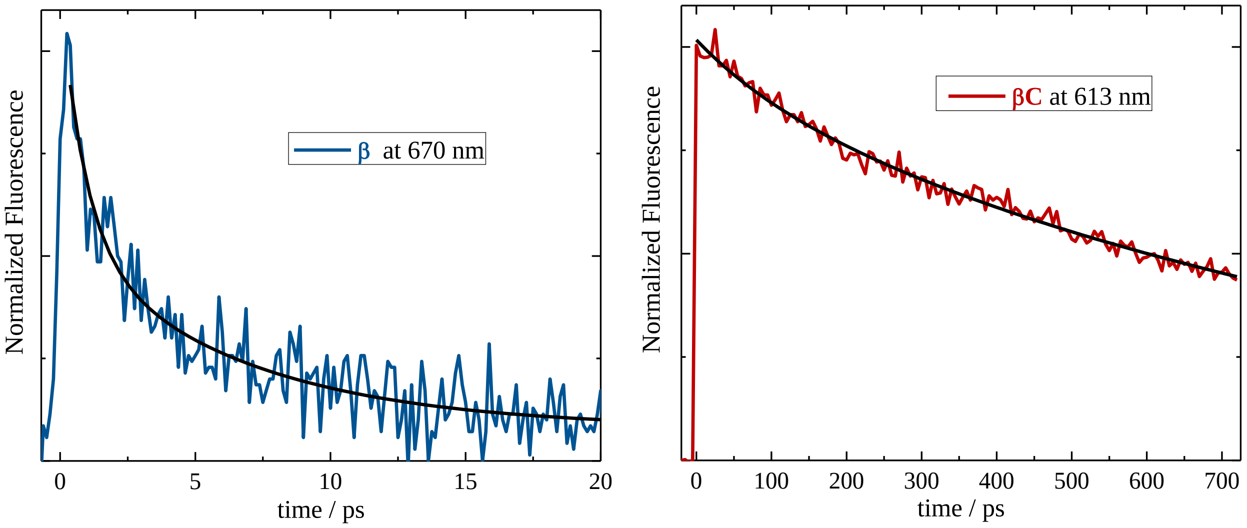

Figure S26. Fluorescence decay kinetics recorded for compounds **β** and **βC** in chloroform by femtosecond resolved FUC, together with their poly-exponential fittings (black).

Table S5. Lifetimes (τ) and pre−exponential factors (A) obtained by fitting the FUC kinetics.

Compound	$A_{FUC,1}$	$\tau_{FUC,1}$ / ps	$A_{FUC,2}$	$\tau_{FUC,2}$ / ps	$A_{FUC,3}$	$\tau_{FUC,3}$ / ps
**β**	0.60	1.0	0.46	6.0	–	–[b]
**βC**	−0.20[a]	0.20[a]	0.11	110	0.90	1000[c]
assignment		*Solv. / ICT*		*VC / SR*		S_1

[a] Rise time obtained from fitting of the fluorescence kinetics acquired on a 3 ps time window (see Figure S22 left). [b] The fact that the third component (τ_3 in Table S4) is not revealed is likely due to acquisition at 670 nm, in the blue part of the emission spectrum, where time resolved red shift of the emission spectra accompanying relaxation is primarily detected. Acquisition at the emission maximum was indeed prevented by the extremely red shifted fluorescence of **β**. [c] Corresponds to the lifetime obtained with the single photon counting measurements (1.33 ns) with the latter being a better evaluation for the decay time of this long living component.

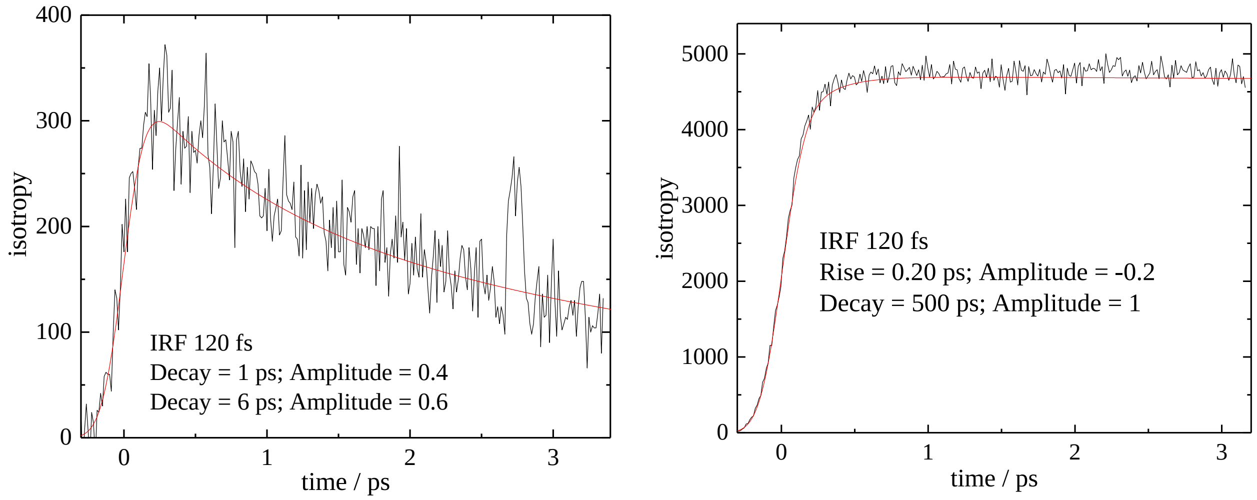

Figure S27. Fluorescence kinetics for **β** at 670 nm (left) and **βC** at 613 nm (right) in chloroform; best fit to a bi−exponential function is also shown.

9. Quantum Chemical Simulations

Details about intersystem crossing rate calculations

The first excited triplet state (T_1) geometry of the compound **β** and **βC** was obtained with time−dependent density functional theory (TDDFT). The same functional and basis sets (B3LYP and 6−31G*) used to locate the ground state geometry were employed for the geometry optimization. Single−point energy was refined using the system−dependent, nonempirically tuned version of long−range corrected functional *ω*B97X−D with the optimal *ω* values. (You can find the optimal *ω* values in the main text.) The medium effect was included using polarizable continuum model with the dielectric constant of 4.31 for chloroform. The first excited singlet (S_1) geometry was used to calculate the spin–orbit coupling. All the quantum chemical simulations were conducted using Q–Chem 5.0.
The rate constant of intersystem crossing (ISC) was estimated via Fermi's Golden rule.[36,37]

$$k_{\mathrm{ISC}} = \frac{2\pi}{\hbar}\rho_{\mathrm{FC}}|\langle \mathrm{S}_1|H_{SO}|\mathrm{T}_1\rangle|^2$$

Where, $\langle \mathrm{S}_1|H_{SO}|\mathrm{T}_1\rangle$ is the spin−orbit coupling element between S_1 and T_1, ρ_{FC} denotes the Franck−Condon−weighted density of states, and $\hbar$ is the reduced Planck constant of 6.582×10^{-16} eVs. ρ_{FC} is evaluated with Marcus−Levich−Jortner theory.[38,39]^{39,40}^{39,40}^{11,12}^{11,12}^{7,8}^{7,8}

$$\rho_{\mathrm{FC}} = \frac{1}{\sqrt{4\pi\lambda_{\mathrm{M}}k_{\mathrm{B}}T}}\sum_{n=0}^{\infty}\exp(-S)\frac{S^n}{n!}\exp[-\frac{(\Delta E_{\mathrm{ST}} + n\hbar\omega_{\mathrm{eff}} + \lambda_{\mathrm{M}})^2}{4\pi\lambda_{\mathrm{M}}k_{\mathrm{B}}T}]$$

Where, λ_{M} is the Marcus reorganization energy associated with the intermolecular and intramolecular low−frequency vibrations, k_{B} is for Boltzmann constant of 8.6173×10^{-5} eV/K, T is the temperature (in this study, 298.15 K), $\hbar\omega_{\mathrm{eff}}$ represents the effective energy of a mode representing the nonclassical high−frequency intramolecular vibrations, and ΔE_{ST} is the adiabatic energy difference between S_1 and T_1. Huang−Rhys factor associated with these modes are given as S.
One recent computational study on thermally activated delayed fluorescence (TADF) mechanism discussed the rate of reverse ISC within the same framework used in this work.[41] The researchers from the same group computed the contribution of nonclassical intramolecular vibrations, and estimated the Marcus reorganization energy due to low−frequency intramolecular vibrations and the medium−induced relaxation effects. In addition, they assumed the Huang−Rhys factors can be neglected without significant changes to the results for large molecules.[39,42]
The numerical values of each property to predict the rate of ISC are given in Table S6.

Table S6. Properties used to predict the rate of ISC.

	**β**	**βC**
λ (eV)	0.244	0.424
Spin–orbit coupling (cm^{-1})	2.704	0.257
ΔE_{ST} (eV)	−0.704	−0.595
Rate of ISC (s^{-1})	8.44×10^5	1.35×10^7

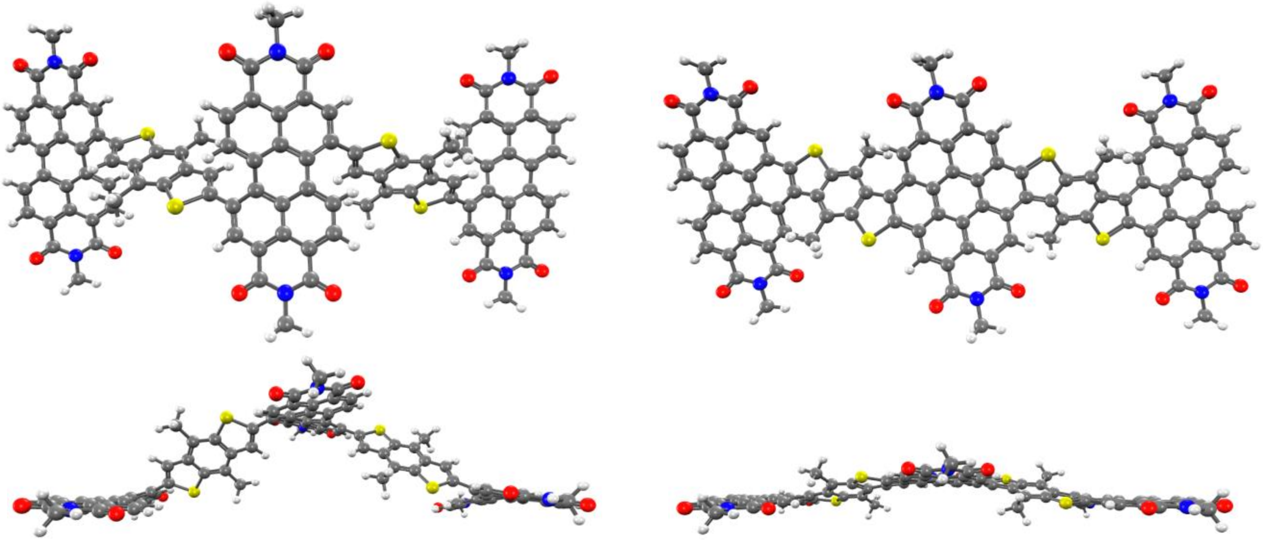

β **βC**

Figure S28. Ground state (S_0) optimized geometry computed for the trimers (color scheme; Hydrogen—white, carbon—black, nitrogen—blue, oxygen—red, sulfur—yellow).

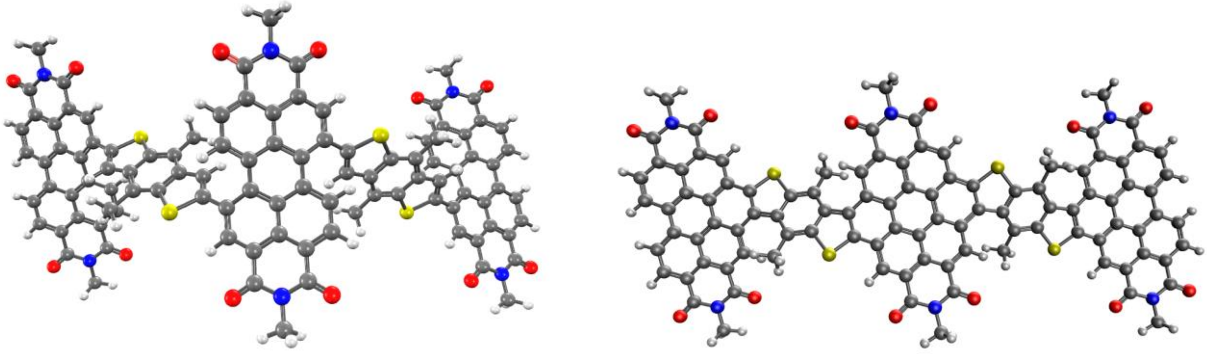

β **βC**

Figure S29. Excited state (T_1) optimized geometry computed for the trimers.

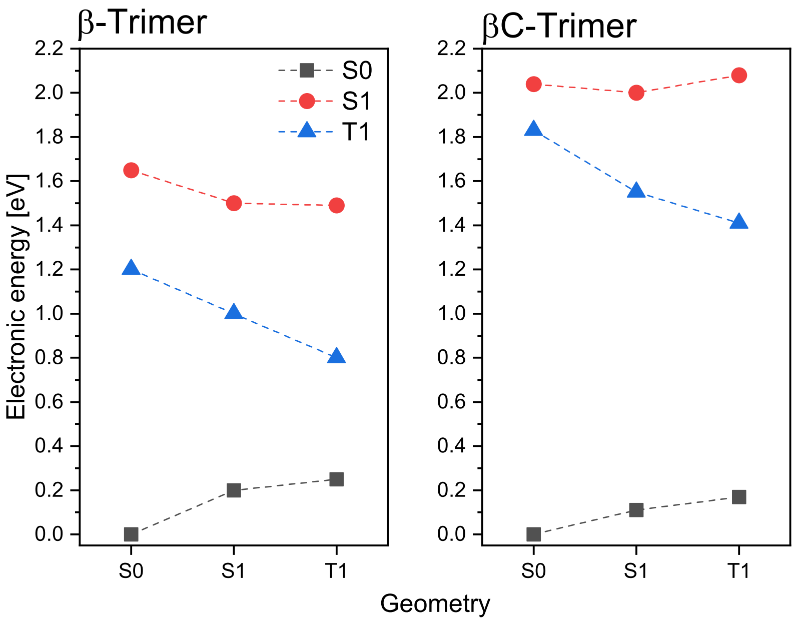

eV	β			βC		
**Geometry/ Electronic state**	S_0	S_1	T_1	S_0	S_1	T_1
$\mathbf{S_0}$	0.00	0.20	0.25	0.00	0.11	0.17
$\mathbf{S_1}$	1.65	1.50	1.49	2.04	2.00	2.08
$\mathbf{T_1}$	1.20	1.00	0.80	1.83	1.55	1.41

Figure S30. Singlet fission energetics for **β** (left) and **βC** (right); energies are given in eV.

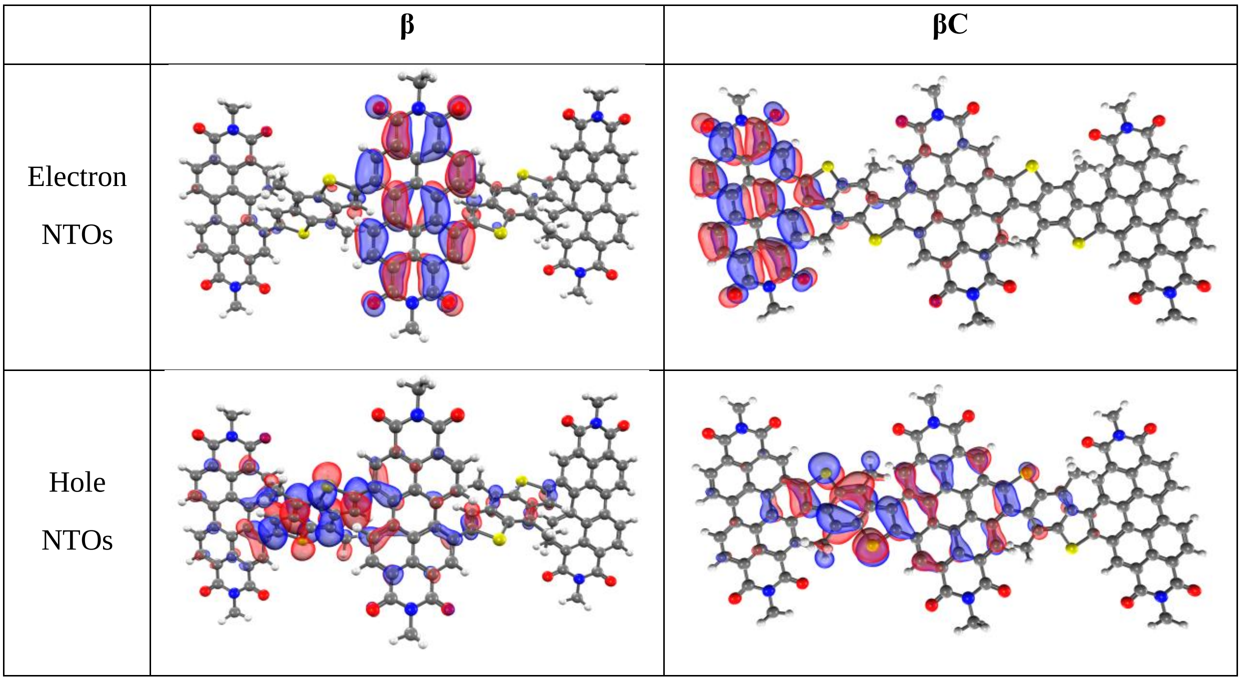

Figure S31. Natural transition orbitals for the S_1 geometry ($S_1 \rightarrow S_0$ transition) of compounds **β** and **βC** (isodensity=0.05. Color scheme; Hydrogen—white, carbon—black, nitrogen—blue, oxygen—red, sulfur—yellow).

	β	βC
Electron NTOs	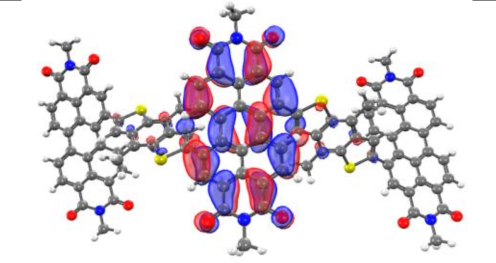	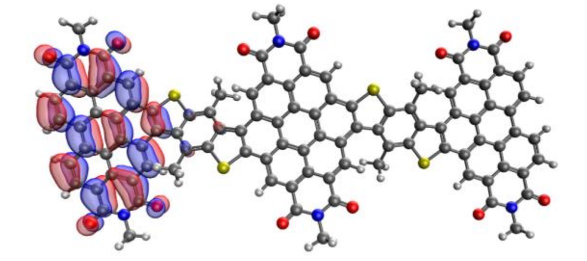
Hole NTOs	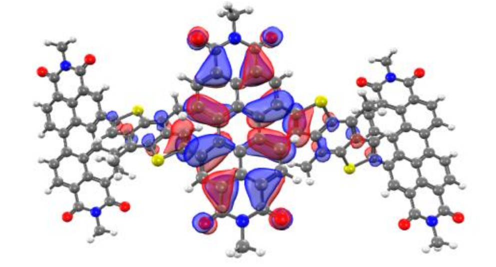	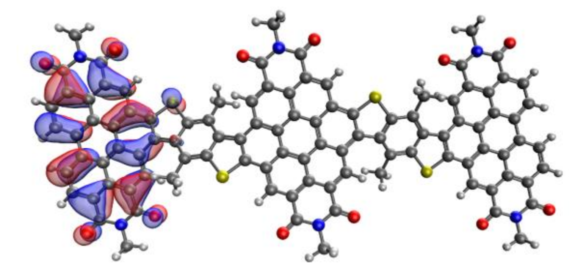

Figure S32. Natural transition orbitals for the T_1 geometry ($S_0 \rightarrow T_1$ transition) for the trimers.

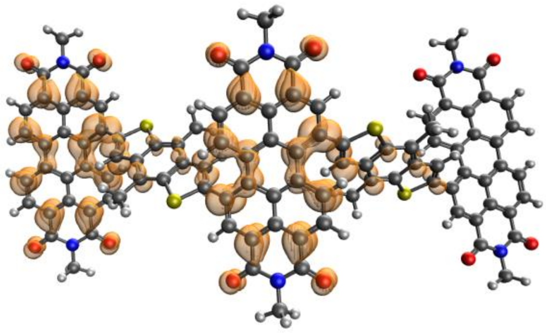

β

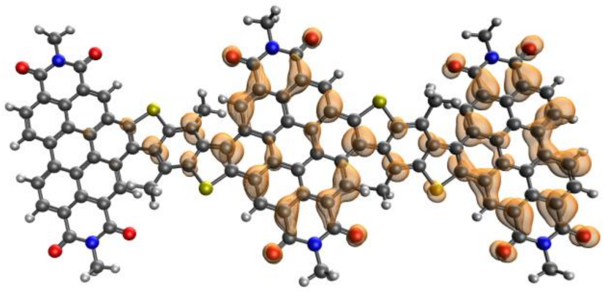

βC

Figure S33. Spin density of quintet state structure for the trimers (isodensity = 0.05. Color scheme; Hydrogen—white, carbon—black, nitrogen—blue, oxygen—red, sulfur—yellow).

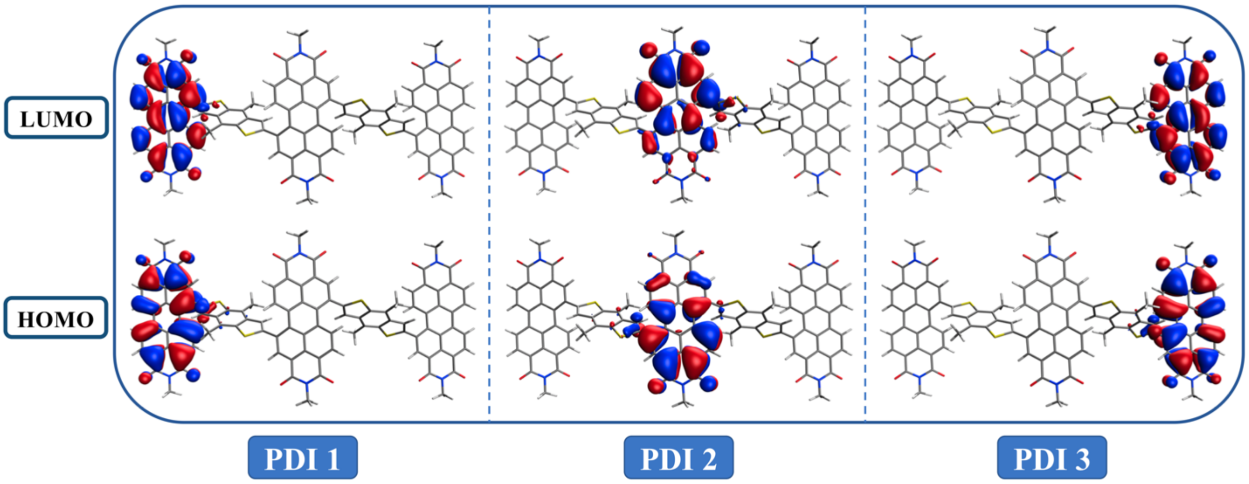

Figure S34. Frontier orbitals considered in RAS-SF calculations for **β** compound.

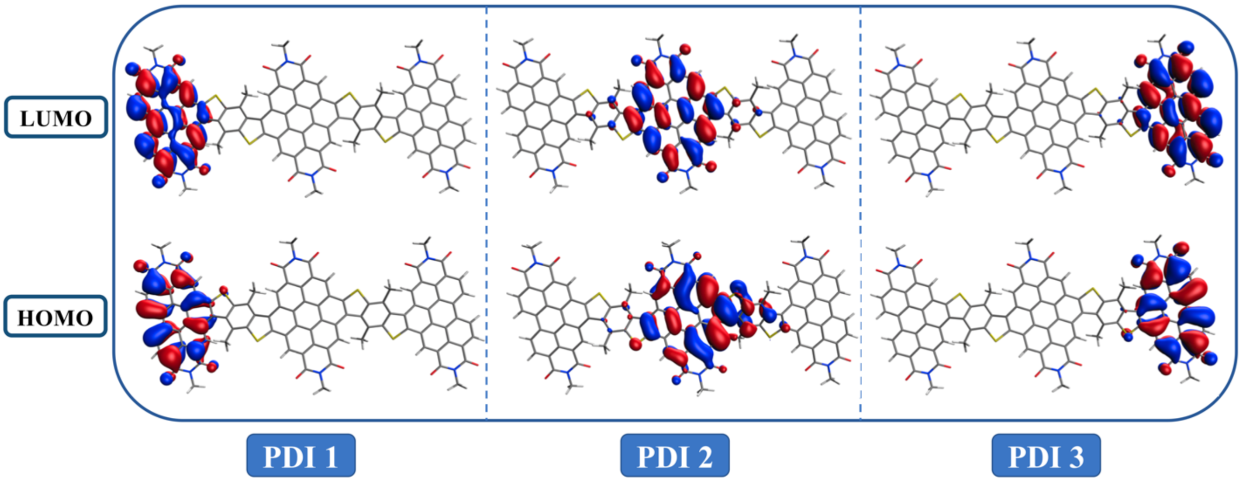

Figure S35. Frontier orbitals considered in RAS-SF calculations for **βC** compound.

Table S7. Energy levels estimated by 3SF-RAS based on the S_1 geometries of each chromophore (S: Singlet, T: Triplet, Q: Quintet, ME: Multiexciton. Energies are given in eV).

β		βC	
State	Energy	State	Energy
S_0	0.000	S_0	0.000
T_1	1.960	T_1	2.371
T_2	1.962	T_2	2.371
T_3	1.967	T_3	2.591
S_1	3.452	S_1	3.683
S_2	3.568	S_2	4.100
S_3	3.586	S_3	4.106
S_4, ME (^{1}TT)	3.940	T_4	4.187
T_4	3.942	T_5	4.335
S_5, ME (^{1}TT)	3.946	T_6	4.340
Q_1	3.946	S_4	4.596
T_5	3.948	S_5	4.604
Q_2	3.952	T_7	4.721
S_6, ME (^{1}TT)	3.955	T_8	4.732
T_6	3.955	T_9	4.785
Q_3	3.955	Q_1	4.785
		S_6, ME (^{1}TT)	4.785
		S_7	4.812
		S_8, ME (^{1}TT)	4.916
		T_{10}	4.917
		S_9, ME (^{1}TT)	4.919
		T_{11}	4.925
		S_{10}	4.962
		Q_2	4.982
		Q_3	4.982

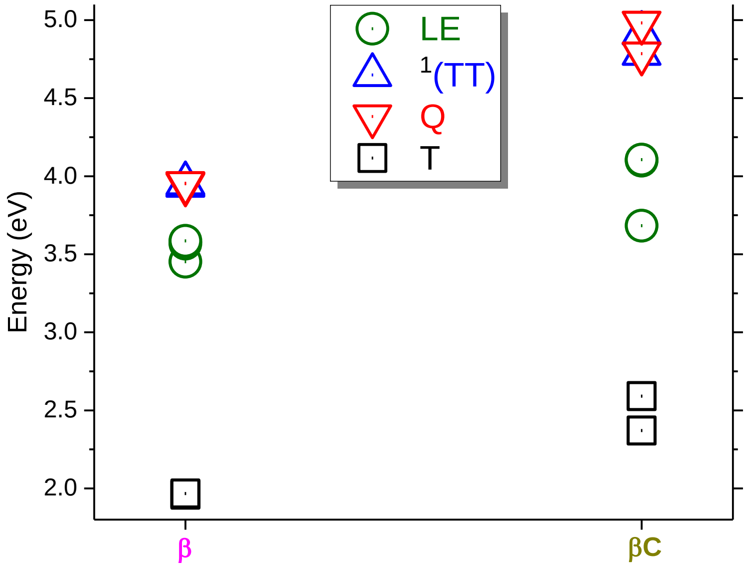

Figure S36. SF relevant energetics for **β** and **βC** Trimers showing interaction energies.
It should be noted that the LE (S_1) and T_1 energies are overestimated in SF-RAS calculations relative to TD-DFT calculations.

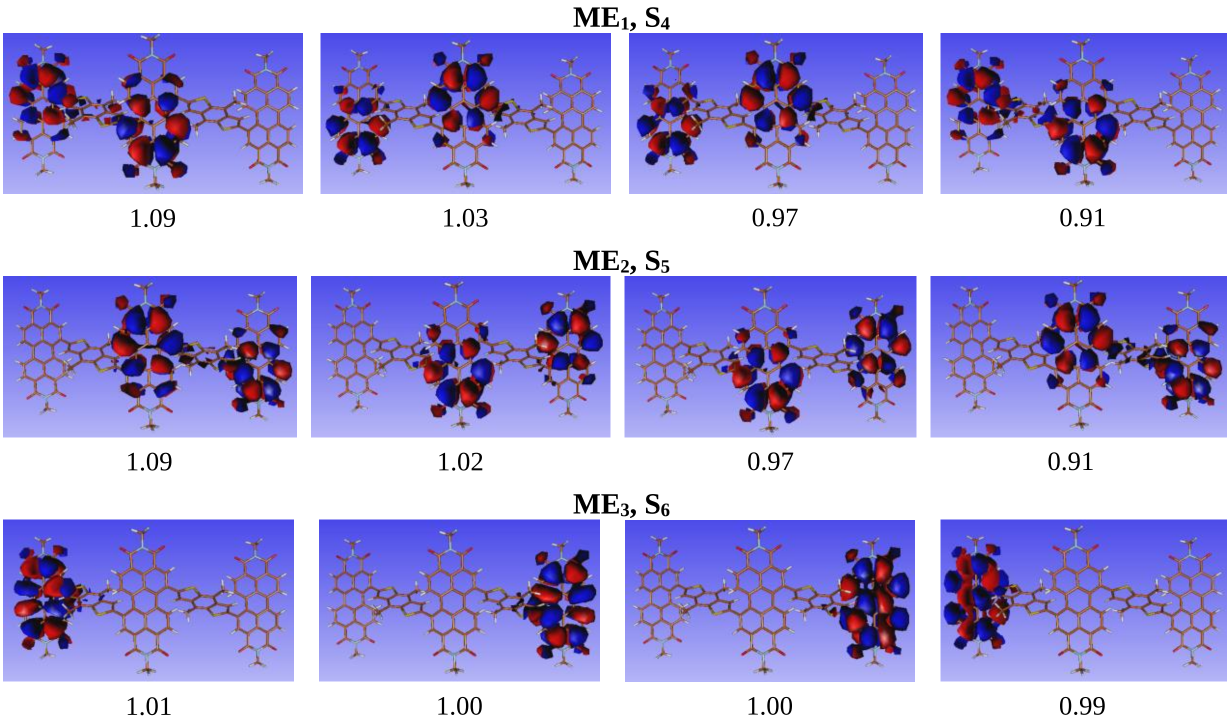

Figure S37. Natural Orbitals for the Multiexcitonic States of the **β** trimer together with their occupation numbers.

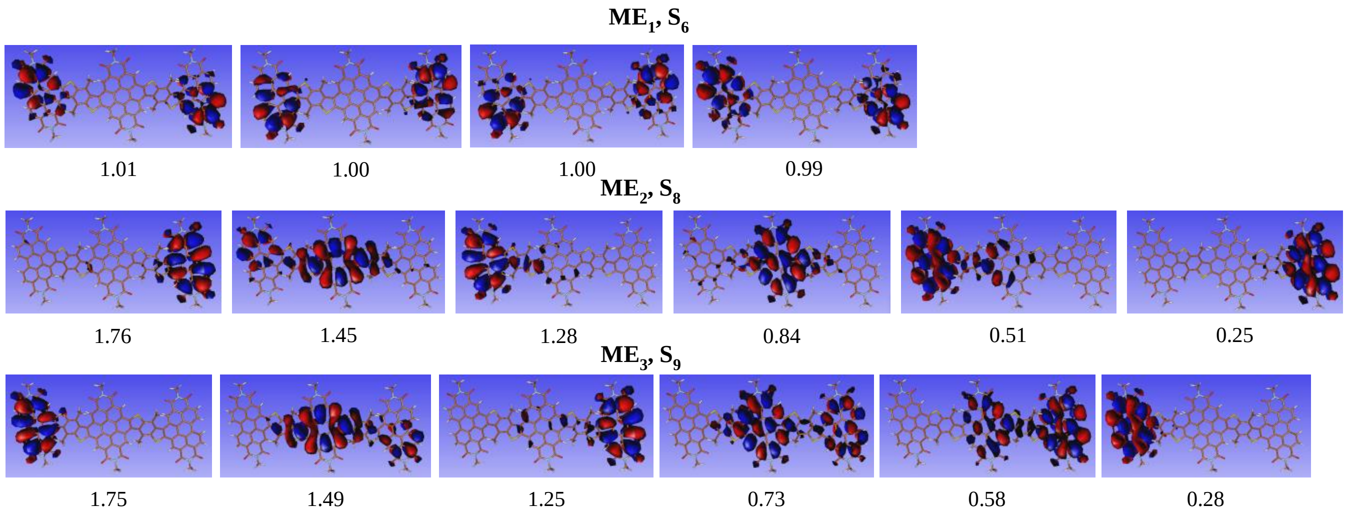

Figure S38. Natural Orbitals for the Multiexcitonic States of the **βC** trimer together with their occupation numbers.

Table S8. Relevant thermodynamic quantities (eV) for **β** and **βC** at 298 K computed following Krylov et al.

	β	βC
$E_{stt} = 2 \times E(T_1) - E(S_1)$	–0.20	+0.59
$E_b = E(^5TT) - E(^1TT)$	+0.006	+0.066
$H_1 = E_{stt} - E_b$	–0.206	+0.524
$H_2 = E_{stt}$	–0.20	+0.59
$TS_1 = k_B T \ln \Omega_1$	+0.028	0
$TS_2 = k_B T \ln \Omega_2$	+0.028	0
$G_1 = E_{stt} - E_b - TS_1$	–0.234	+0.524
$G_2 = E_{stt} - TS_2$	–0.228	+0.59
$\boldsymbol{\Delta H_{TOT} = \Delta H_1 + \Delta H_2 = E_{stt}}$	**–0.20**	**+0.59**
$\boldsymbol{T\Delta S_{TOT} = T\Delta S_1 + T\Delta S_2}$	**+0.028**	**0**
$\boldsymbol{\Delta G_{TOT} = \Delta G_1 + \Delta G_2}$	**–0.228**	**+0.59**

1 is the first step of singlet fission (from state 0, S_1, to state 1, ^1TT); 2 is the second step of triplet separation (from state 1, ^1TT, to state 2, T_1); for **β**, $\Omega_1=\Omega_2=3$, all the three double triplets are energetically close and accessible; for **βC**, $\Omega_1=\Omega_2=1$, only one double triplet state is energetically accessible; $\Delta\chi_1=\chi_1-\chi_0$ and $\Delta\chi_2=\chi_2-\chi_1$ where χ is H, S or G; $k_BT=0.026$ eV at 298 K.

10. ^{1}H and ^{13}C NMR of the compounds

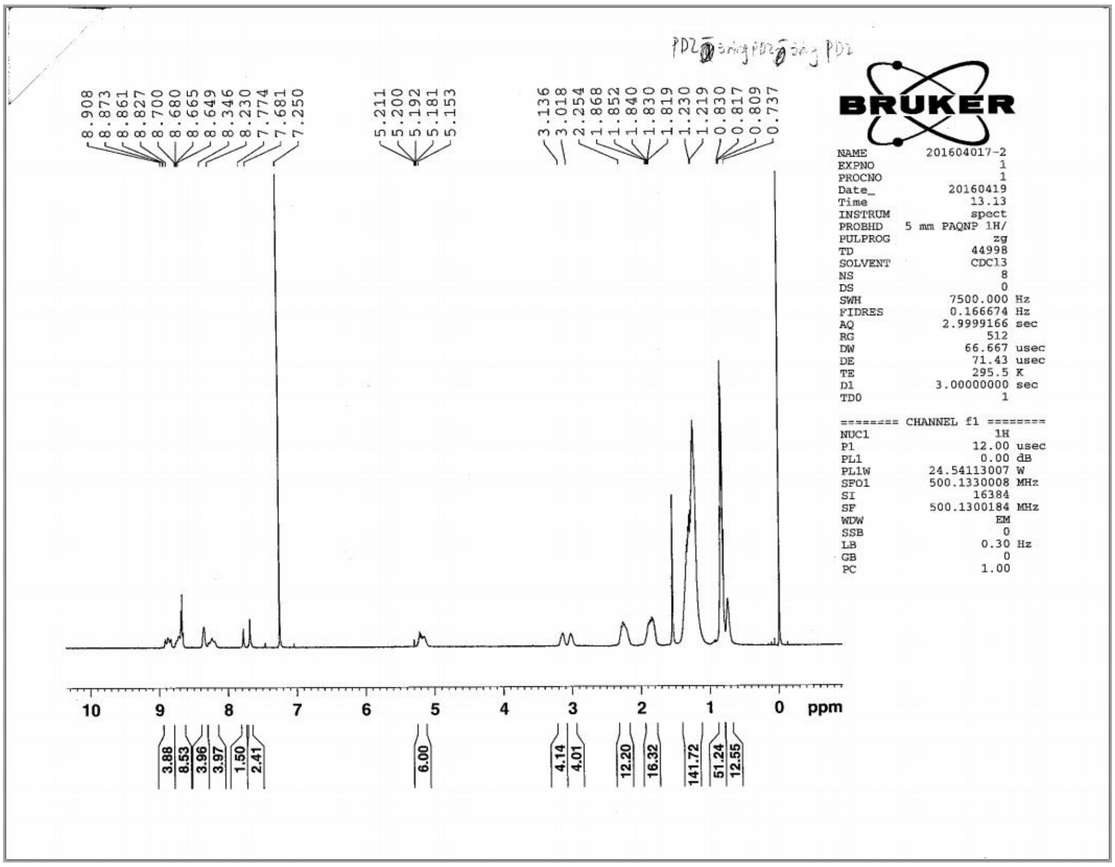

Figure S39. ^{1}H NMR of compound **β**

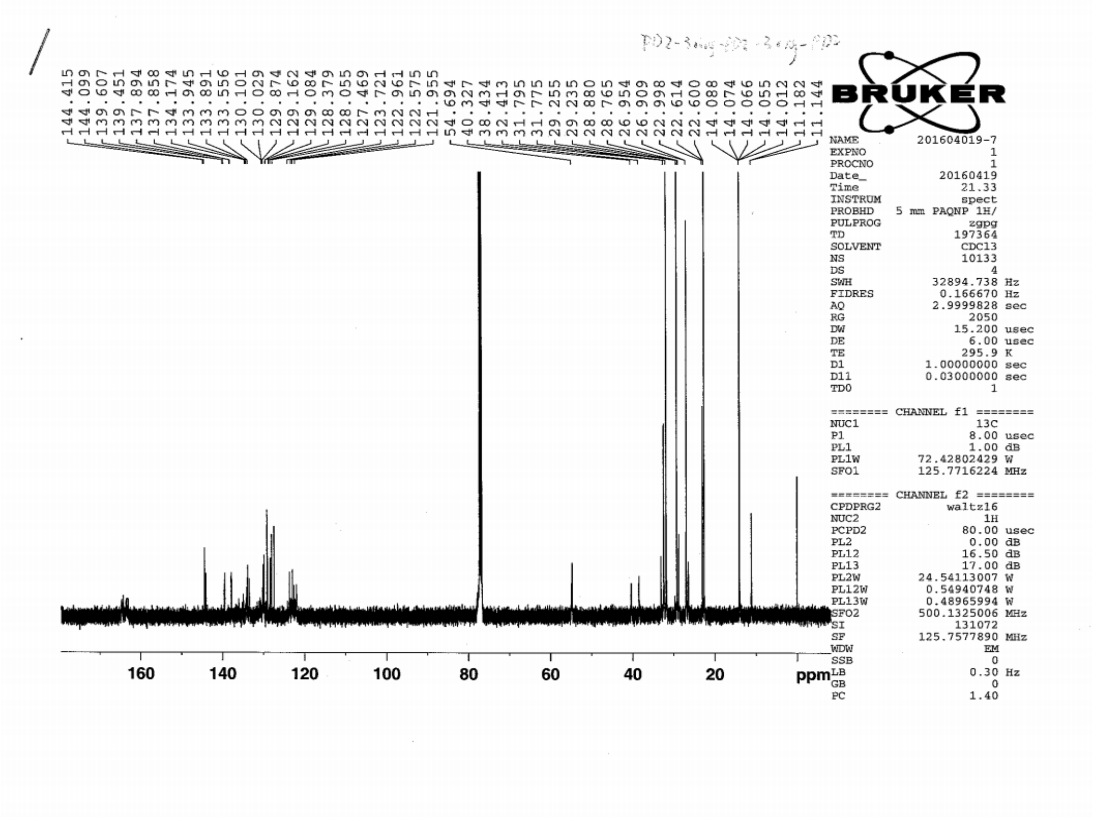

Figure S40. ^{13}C NMR of compound **β**

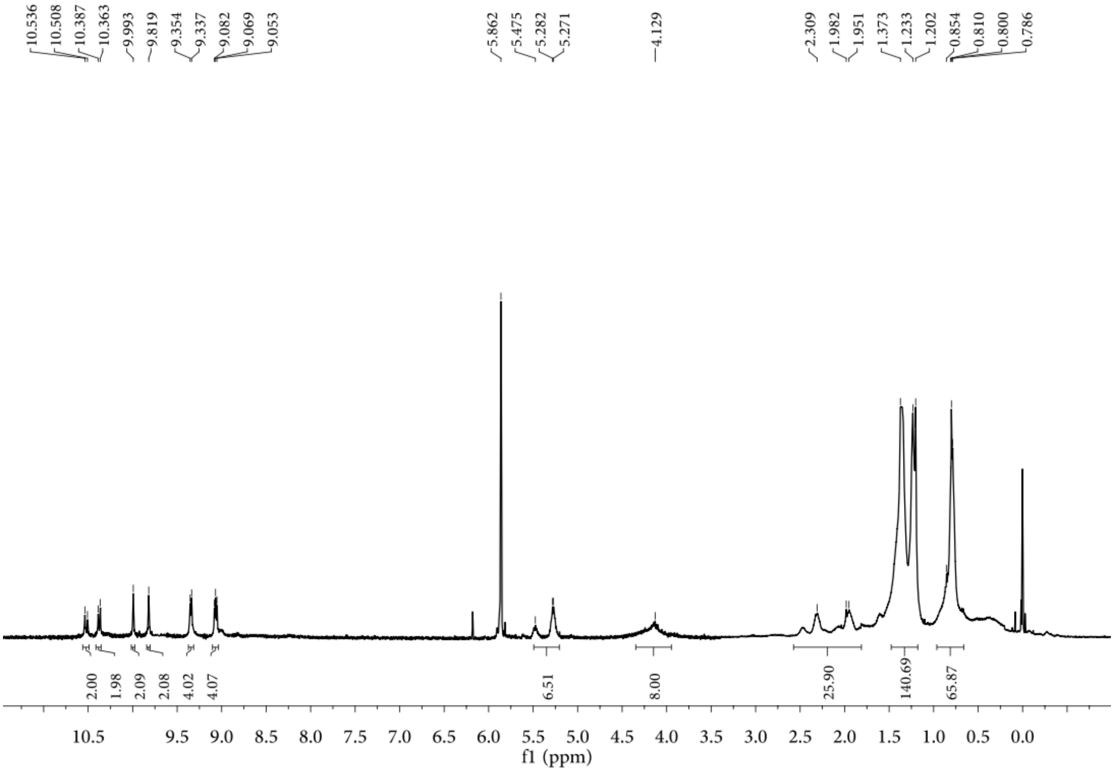

Figure S41. ^{1}H NMR of compound **βC**

References:

(1) Cai, Z.; Vázquez, R. J.; Zhao, D.; Li, L.; Lo, W. Y.; Zhang, N.; Wu, Q.; Keller, B.; Eshun, A.; Abeyasinghe, N.; et al. Two Photon Absorption Study of Low-Bandgap, Fully Conjugated Perylene Diimide-Thienoacene-Perylene Diimide Ladder-Type Molecules. *Chem. Mater.* **2017**, *29* (16), 6726–6732.

(2) Guan, J.; Tomobe, K.; Madu, I.; Goodson, T.; Makhal, K.; Trinh, M. T.; Rand, S. C.; Yodsin, N.; Jungsuttiwong, S.; Laine, R. M. Photophysical Properties of Partially Functionalized Phenylsilsesquioxane: [RSiO1.5]7[Me/NPrSiO1.5] and [RSiO1.5]7[O0.5SiMe3]3 (R = 4-Me/4-CN-Stilbene). Cage-Centered Magnetic Fields Form under Intense Laser Light. *Macromolecules* **2019**, *52*, 4008–4019.

(3) Jobin Yvon. *A Guide to Recording Fluorescence Quantum Yields Introduction:*; 2014; Vol. 15.

(4) Kubin, R. F.; Fletcher, A. N. Fluorescence Quantum Yields of Some Rhodamine Dyes. *J. Lumin.* **1982**, *27* (4), 455–462.

(5) Keller, B.; McLean, A.; Kim, B. G.; Chung, K.; Kim, J.; Goodson, T. Ultrafast Spectroscopic Study of Donor-Acceptor Benzodithiophene Light Harvesting Organic Conjugated Polymers. *J. Phys. Chem. C* **2016**, *120* (17), 9088–9096.

(6) Villabona-Monsalve, J. P.; Varnavski, O.; Palfey, B. A.; Goodson, T. Two-Photon Excitation of Flavins and Flavoproteins with Classical and Quantum Light. *J. Am. Chem. Soc.* **2018**, *140* (44), 14562–14566.

(7) Xu, C.; Webb, W. W. Measurement of Two-Photon Excitation Cross Sections of Molecular Fluorophores with Data from 690 to 1050 Nm. *J. Opt. Soc. Am. B* **1996**, *13* (3), 481.

(8) Madu, I. K.; Muller, E. W.; Kim, H.; Shaw, J.; Burney-Allen, A.; Zimmerman, P. M.; Jeffries-EL, M.; Goodson III, T. Heteroatom and Side Chain Effects on the Optical and Photo-Physical Properties: Ultrafast and Nonlinear Spectroscopy of New Naphtho[1,2-B:5,6-B′]Difuran Donor Polymers. *J. Phys. Chem. C* **2018**, acs.jpcc.8b03914.

(9) Carlotti, B.; Cai, Z.; Kim, H.; Sharapov, V.; Madu, I. K.; Zhao, D.; Chen, W.; Zimmerman, P. M.; Yu, L.; Goodson, T. Charge Transfer and Aggregation E Ff Ects on the Performance of Planar vs Twisted Nonfullerene Acceptor Isomers for Organic Solar Cells. *Chem. Mater.* **2018**, *30*, 4263–4276.

(10) Vázquez, R. J.; Kim, H.; Zimmerman, P. M.; Goodson, T. Using Ultra-Fast Spectroscopy to Probe the Excited State Dynamics of a Reported Highly Efficient Thermally Activated Delayed Fluorescence Chromophore. *J. Mater. Chem. C* **2019**, *7* (14), 4210–4221.

(11) Carmichael, I.; Hug, G. L. Triplet-Triplet Absorption Spectra of Organic Molecules in Condensed Phases. *J. Phys. Chem. Ref. Data* **1986**, *15* (1), 1–250.

(12) Bensasson, R.; Dawe, E. A.; Long, D. A.; Land, E. J. Singlet → Triplet Intersystem Crossing Quantum Yields of Photosynthetic and Related Polyenes. *J. Chem. Soc. Faraday Trans. 1 Phys. Chem. Condens. Phases* **1977**, *73*, 1319.

(13) Murov, S.; Carmichael, I.; Hug, G. L. *Handbook of Photochemistry*, second.; MARCEL DEKKER, INC.: New York, 1993.

(14) Cogdell, R. J.; Land, E. J.; Truscott, T. G. The Triplet Extinction Coefficients of Some Bacterial Carotenoids. *Photochem. Photobiol.* **1983**, *38* (6), 723–725.

(15) Amouyal, E.; Bensasson, R.; Land, E. J. Triplet States of Ubiquinone Analogs Studied By Ultraviolet and Electron Nanosecond Irradiation. *Photochem. Photobiol.* **1974**, *20* (5), 415–422.

(16) Nielsen, B. R.; Jørgensen, K.; Skibsted, L. H. Triplet-Triplet Extinction Coefficients, Rate Constants of Triplet Decay and Rate Constants of Anthracene Triplet Sensitization by Laser Flash Photolysis of Astaxanthin, β-Carotene, Canthaxanthin and Zeaxanthin in Deaerated Toluene at 298 K. *J. Photochem. Photobiol. A Chem.* **1998**, *112* (2–3), 127–133.

(17) Varnavski, O.; Abeyasinghe, N.; Aragó, J.; Serrano-Pérez, J. J.; Ortí, E.; López Navarrete, J. T.; Takimiya, K.; Casanova, D.; Casado, J.; Goodson, T. High Yield Ultrafast Intramolecular Singlet Exciton Fission in a Quinoidal Bithiophene. *J. Phys. Chem. Lett.* **2015**, *6* (8), 1375–1384.

(18) Zhang, J.; Sulaiman, S.; Madu, I. K.; Laine, R. M.; Goodson, T. Ultrafast Excited-State Dynamics of Partially and Fully Functionalized Silsesquioxanes. *J. Phys. Chem. C* **2019**, *123* (8), 5048–5060.

(19) Taylor, E. L.; Metcalf, K. J.; Carlotti, B.; Lai, C. T.; Modica, J. A.; Schatz, G. C.; Mrksich, M.; Goodson, T. Long-Range Energy Transfer in Protein Megamolecules. *J. Am. Chem. Soc.* **2018**, *140* (46), 15731–15743.

(20) Adegoke, O. O.; Jung, I. H.; Orr, M.; Yu, L.; Goodson, T. Effect of Acceptor Strength on Optical and Electronic Properties in Conjugated Polymers for Solar Applications. *J. Am. Chem. Soc.* **2015**, *137* (17), 5759–5769.

(21) Chai, J.-D.; Head-Gordon, M. Long-Range Corrected Hybrid Density Functionals with Damped Atom–Atom Dispersion Corrections. *Phys. Chem. Chem. Phys.* **2008**, *10* (44), 6615.

(22) Chai, J. Da; Head-Gordon, M. Systematic Optimization of Long-Range Corrected Hybrid Density Functionals. *J. Chem. Phys.* **2008**, *128* (8).

(23) Zheng, Z.; Brédas, J.-L.; Coropceanu, V. Description of the Charge Transfer States at the Pentacene/C_{60} Interface: Combining Range-Separated Hybrid Functionals with the Polarizable Continuum Model. *J. Phys. Chem. Lett.* **2016**, *7* (13), 2616–2621.

(24) Farag, M. H.; Krylov, A. I. Singlet Fission in Perylenediimide Dimers. *J. Phys. Chem. C* **2018**, *122* (45), 25753–25763.

(25) Korovina, N. V.; Das, S.; Nett, Z.; Feng, X.; Joy, J.; Haiges, R.; Krylov, A. I.; Bradforth, S. E.; Thompson, M. E. Singlet Fission in a Covalently Linked Cofacial Alkynyltetracene Dimer. *J. Am. Chem. Soc.* **2016**, *138* (2), 617–627.

(26) Casanova, D. Electronic Structure Study of Singlet Fission in Tetracene Derivatives. *J. Chem. Theory Comput.* **2014**, *10* (1), 324–334.

(27) Zimmerman, P. M.; Musgrave, C. B.; Head-Gordon, M. A Correlated Electron View of Singlet Fission. *Acc. Chem. Res.* **2013**, *46* (6), 1339–1347.

(28) Zimmerman, P. M.; Bell, F.; Casanova, D.; Head-Gordon, M. Mechanism for Singlet Fission in Pentacene and Tetracene: From Single Exciton to Two Triplets. *J. Am. Chem. Soc.* **2011**, *133* (49), 19944–19952.

(29) Kolomeisky, A. B.; Feng, X.; Krylov, A. I. A Simple Kinetic Model for Singlet Fission: A Role of Electronic and Entropic Contributions to Macroscopic Rates. *J. Phys. Chem. C* **2014**, *118* (10), 5188–5195.

(30) Feng, X.; Casanova, D.; Krylov, A. I. Intra- and Intermolecular Singlet Fission in Covalently Linked Dimers. *J. Phys. Chem. C* **2016**, *120* (34), 19070–19077.

(31) Piland, G. B.; Burdett, J. J.; Dillon, R. J.; Bardeen, C. J. Singlet Fission: From Coherences to Kinetics. *J. Phys. Chem. Lett.* **2014**, *5* (13), 2312–2319.

(32) Chien, A. D.; Zimmerman, P. M. Recovering Dynamic Correlation in Spin Flip Configuration Interaction through a Difference Dedicated Approach. *J. Chem. Phys.* **2017**, *146* (1).

(33) Shao, Y.; Gan, Z.; Epifanovsky, E.; Gilbert, A. T. B.; Wormit, M.; Kussmann, J.; Lange, A. W.; Behn, A.; Deng, J.; Feng, X.; et al. Advances in Molecular Quantum Chemistry Contained in the Q-Chem 4 Program Package. *Mol. Phys.* **2015**, *113* (2), 184–215.

(34) Margulies, E. A.; Miller, C. E.; Wu, Y.; Ma, L.; Schatz, G. C.; Young, R. M.; Wasielewski, M. R. Enabling Singlet Fission by Controlling Intramolecular Charge Transfer in π-Stacked Covalent Terrylenediimide Dimers. *Nat. Chem.* **2016**, *8* (12), 1120–1125.

(35) Ramanan, C.; Smeigh, A. L.; Anthony, J. E.; Marks, T. J.; Wasielewski, M. R. Competition between

Singlet Fission and Charge Separation in Solution-Processed Blend Films of 6,13-Bis(Triisopropylsilylethynyl)Pentacene with Sterically-Encumbered Perylene-3,4:9,10-Bis(Dicarboximide)S. *J. Am. Chem. Soc.* **2012**, *134* (1), 386–397.

(36) Robinson, G. W.; Frosch, R. P. Electronic Excitation Transfer and Relaxation. *J. Chem. Phys.* **1963**, *38* (5), 1187–1203.

(37) Lawetz, V.; Orlandi, G.; Siebrand, W. Theory of Intersystem Crossing in Aromatic Hydrocarbons. *J. Chem. Phys.* **1972**, *56* (8), 4058–4072.

(38) Brédas, J.-L.; Beljonne, D.; Coropceanu, V.; Cornil, J. Charge-Transfer and Energy-Transfer Processes in π-Conjugated Oligomers and Polymers: A Molecular Picture. *Chem. Rev.* **2004**, *104* (11), 4971–5004.

(39) Schmidt, K.; Brovelli, S.; Coropceanu, V.; Beljonne, D.; Cornil, J.; Bazzini, C.; Caronna, T.; Tubino, R.; Meinardi, F.; Shuai, Z.; et al. Intersystem Crossing Processes in Nonplanar Aromatic Heterocyclic Molecules. *J. Phys. Chem. A* **2007**, *111* (42), 10490–10499.

(40) Jean-Luc Brédas, *,†,‡; David Beljonne, †,‡; Veaceslav Coropceanu, † and; Jérôme Cornil†, ‡. Charge-Transfer and Energy-Transfer Processes in π-Conjugated Oligomers and Polymers: A Molecular Picture. **2004**.

(41) Samanta, P. K.; Kim, D.; Coropceanu, V.; Brédas, J. L. Up-Conversion Intersystem Crossing Rates in Organic Emitters for Thermally Activated Delayed Fluorescence: Impact of the Nature of Singlet vs Triplet Excited States. *J. Am. Chem. Soc.* **2017**, *139* (11), 4042–4051.

(42) Beljonne, D.; Shuai, Z.; Pourtois, G.; Bredas, J. L. Spin-Orbit Coupling and Intersystem Crossing in Conjugated Polymers: A Configuration Interaction Description. *J. Phys. Chem. A* **2001**, *105* (15), 3899–3907.
